# Dose–Response Modelling of Resistance Exercise Across Outcome Domains in Strength and Conditioning: A Meta-analysis

**DOI:** 10.1007/s40279-024-02006-3

**Published:** 2024-04-23

**Authors:** Paul Alan Swinton, Brad J. Schoenfeld, Andrew Murphy

**Affiliations:** 1 School of Health Sciences, Robert Gordon University, Garthdee Road, Aberdeen, AB10 7QG UK; 2 Department of Exercise Science and Recreation, CUNY Lehman College, Bronx, NY USA; 3 Greater Western Sydney Giants, Sydney, NSW Australia

## Abstract

**Background:**

Resistance exercise is the most common training modality included within strength and conditioning (S&C) practice. Understanding dose–response relationships between resistance training and a range of outcomes relevant to physical and sporting performance is of primary importance for quality S&C prescription.

**Objectives:**

The aim of this meta-analysis was to use contemporary modelling techniques to investigate resistance-only and resistance-dominant training interventions, and explore relationships between training variables (frequency, volume, intensity), participant characteristics (training status, sex), and improvements across a range of outcome domains including maximum strength, power, vertical jump, change of direction, and sprinting performance.

**Methods:**

Data were obtained from a database of training studies conducted between 1962 and 2018, which comprised healthy trained or untrained adults engaged in resistance-only or resistance-dominant interventions. Studies were not required to include a control group. Standardized mean difference effect sizes were calculated and interventions categorized according to a range of training variables describing frequency (number of sessions per week), volume (number of sets and repetitions performed), overall intensity (intensity of effort and load, categorised as low, medium or high), and intensity of load (represented as % of one-repetition maximum [1RM] prescribed). Contemporary modelling techniques including Bayesian mixed-effects meta-analytic models were fitted to investigate linear and non-linear dose-responses with models compared based on predictive accuracy.

**Results:**

Data from a total of 295 studies comprising 535 groups and 6,710 participants were included with analyses conducted on time points ≤ 26 weeks. The best performing model included: duration from baseline, average number of sets, and the main and interaction effects between outcome domain and intensity of load (% 1RM) expressed non-linearly. Model performance was not improved by the inclusion of participant training status or sex.

**Conclusions:**

The current meta-analysis represents the most comprehensive investigation of dose–response relationships across a range of outcome domains commonly targeted within strength and conditioning to date. Results demonstrate the magnitude of improvements is predominantly influenced by training intensity of load and the outcome measured. When considering the effects of intensity as a % 1RM, profiles differ across outcome domains with maximum strength likely to be maximised with the heaviest loads, vertical jump performance likely to be maximised with relatively light loads (~ 30% 1RM), and power likely to be maximised with low to moderate loads (40–70% 1RM).

**Supplementary Information:**

The online version contains supplementary material available at 10.1007/s40279-024-02006-3.

## Key Points

**Table:** 

Results from the current large-scale meta-analysis demonstrate that intervention duration combined with manipulation of training intensity are the most relevant factors in altering magnitude of improvement following resistance training.
Different % 1RM intensity profiles exist across outcome domains, highlighting the importance of selecting domain-specific loads.
Across the heterogeneous research, training status, sex and training frequency were shown to provide limited predictive capabilities. These factors should still be considered when developing holistic periodised strength and conditioning programs with further research required.

## Introduction

Resistance exercise is established as one of the most effective training modalities within strength and conditioning (S&C) [1, 2]. Researchers have shown that improvements in strength and power can be transferred to a range of important activities associated with sports performance including sprinting and jumping [1, 3, 4]. Results from a recent large meta-analysis also highlighted the importance of training specificity, in relation to the imposed demands of training, with the greatest improvements made when matching the training stimulus and the outcomes assessed (e.g., traditional resistance exercise with heavy loads matched with one-repetition maximum (1RM) assessment) [5]. Beyond specificity, appropriate prescription of resistance exercise requires consideration of a range of acute program variables including volume, intensity, frequency and potentially more subtle variables including exercise selection and exercise order [6]. There have been attempts to provide general recommendations of training dose to maximise a range of outcome domains including strength, hypertrophy, power and muscular endurance [6]. Previous researchers have also indicated that the training status of participants quantified as years of resistance training experience may play an important role and interact with training dose [7, 8]. Greater understanding of dose–response relationships across a range of factors including the training modality, outcome domain, participant characteristics (e.g., training status) and length of intervention is key for continued development of resistance exercise and the desire to avoid over- or under-loading.

Several systematic reviews have investigated training dose within S&C, with most focusing on development of strength and hypertrophy [7–17]. Seminal studies conducted by Rhea and colleagues [7–9] were among the first to use meta-analytic techniques to quantify dose–response relationships. Initial research from Rhea et al. [9] incorporated data from 16 studies to compare single versus multiple sets and concluded that performance of three sets was more effective than a single set. Follow-up research was conducted by the authors by (1) substantially increasing the number of studies included in the analysis [7], and (2) focusing on higher-level athletes [8]. The large-scale meta-analysis conducted by Rhea et al. [7] included data from 140 studies and provided further support for the superiority of multiple sets, with four sets per muscle group concluded to produce the greatest improvements [7]. The results from the comprehensive analysis indicated different dose–response relationships for trained and untrained participants, with a higher intensity of load (80% 1RM) and a frequency of 2 days per week judged most effective for trained participants; and lower intensity of load (60% 1RM) and a frequency of 3 days per week judged most effective for untrained participants. In the follow-up meta-analysis restricted to 37 studies including competitive athletes, Peterson et al. [8] concluded higher volumes (eight sets per muscle group) and intensity of load intensities (85% 1RM) resulted in the largest effects with no differences found between frequencies of 2 or 3 days per week. Collectively, the work from Rhea and colleagues synthesised results from almost 200 studies and confirmed the existence of dose–response relationships for the development of strength and the likely moderation by participant characteristics including training status [18].

The most recent meta-analyses investigating dose–response relationships in S&C have tended to focus on the manipulation of a smaller number of program variables and restricting analyses to more homogeneous studies [10–17]. Meta-analyses from Grgic et al. [12] and Ralston et al. [13] investigated the effects of manipulating training frequency on strength improvements and incorporated data from 22 studies directly comparing frequencies of 1–4 days per week, and from 12 studies directly comparing frequencies of 1–3 days per week, respectively. Grgic et al. [12] concluded that higher training frequencies resulted in greater improvements in strength, but that these increases appeared to be primarily mediated through increased weekly volume. Similar conclusions were presented by Ralston et al. [13] showing that when resistance volume was equated across low (1 day week^−1^), medium (2 days week^−1^) or high (≥ 3 days week^−1^) frequencies, similar increases in strength were obtained for both isolation and multi-joint exercises. Restricting analyses to trained athletes, Cuthbert et al. [17] identified no differences in improvements to either lower (effect size: 0.06; CI – 0.20, 0.32) or upper (effect size: 0.09; CI – 0.17, 0.35) body strength when comparing training frequencies during competitive periods.

Another smaller and more focused meta-analysis was conducted by Schoenfeld et al. [15] comparing low-load (≤ 60% 1RM) versus high-load (> 60% 1RM) resistance training. The meta-analysis included 21 relatively homogeneous studies tending to focus on exercises including the bench press, knee extension and leg press, all performed to momentary muscular failure. Results from the meta-analysis identified greater improvements in 1RM strength with high-load resistance training; however, the transfer of training to isometric strength testing showed marginally greater effects favouring higher versus lower loads (effect size: 0.16; 95% CI – 0.06, 0.37) [15]. Moreover, similar improvements were observed in muscle hypertrophy across conditions. The results presented by Schoenfeld et al. [15] highlight the potential for different dose–response relationships across outcomes routinely targeted in S&C.

Whilst both large meta-analyses comprising heterogeneous studies and smaller, more focussed meta-analyses present different strengths and weaknesses, there is likely a benefit in simultaneously modelling dose–response relations across a broader range of outcome domains than has been investigated previously. The response to any training program is ultimately a complex interaction of a range of variables and participant characteristics; however, large modelling analyses have the potential to identify general trends that provide researchers and practitioners with important information on which to design more specific programs. With this perspective in mind, the purpose of this encompassing meta-analysis was to use contemporary modelling techniques to investigate resistance training interventions and explore relationships between training variables (e.g., frequency, volume, overall intensity, and intensity of load), participant characteristics (training status, sex), and a range of outcome domains including maximum strength, power, vertical jump, change of direction (COD), and sprinting performance, which are key targets in S&C program design.

## Methods

### Overview of Meta-analysis

The meta-analysis was conducted on a database of S&C training studies with analyses restricted to interventions that comprised resistance training only, or combined interventions where resistance training accounted for more than half of the training volume (e.g., resistance combined with plyometrics, speed, COD or power training). The database included information describing outcome variables, participant characteristics, training-dose parameters along with baseline and follow-up means and standard deviations, as has been described elsewhere [1]. The information was used to calculate intervention-only (e.g., non-controlled) effect sizes designed to draw inferences from indirect comparisons. To conduct the meta-analysis, sequential hierarchical models were fitted to account for dependencies (e.g., reporting of multiple outcomes at multiple time points from the same study) and structure within the data (e.g., time points nested in outcomes, nested in studies). Participant, training dose, and intervention characteristics were sequentially added to models to identify the most influential factors whilst monitoring changes caused by underlying associations among the variables. The focus of this meta-analysis was to quantify and describe the general influence of training dose across a range of outcomes commonly investigated in S&C studies.

### Search Strategy and Reporting

The present review was conducted as a follow-up to a larger review that featured a broader range of training interventions [1]. The search for the original review was performed using Embase, Medline, Web of Science, SPORTDiscus and Google Scholar. Hand searching of relevant journals including *Medicine and Science in Sports and Exercise*, the *Journal of Strength and Conditioning Research*, and *Research Quarterly* was also conducted. Database search terms were included to identify various training modes and a range of outcome measures. The following keywords and phrases were combined with Boolean operators; “strength” OR “resistance” OR “sprint” OR “plyometric” OR “exercise” AND “intervention” OR “training” OR “program” OR “programme” AND “1RM” OR “repetition maximum” OR “speed” OR “velocity” OR “power” OR “jump” OR “change of direction” OR “agility” OR “acceleration” OR “rate of force development”. No restriction was placed on the date of the study, with searching conducted in January 2018. Reporting of this review was guided by the Preferred Reporting Items for Systematic Reviews and Meta-Analyses (PRISMA) 2020 [19] statement, with the checklist included for transparency (Online Supplementary Material (OSM) 1). Risk of bias assessment was not conducted.

### Inclusion Criteria and Data Extraction

Inclusion and exclusion criteria for the current meta-analysis were set to include as many relevant S&C training studies as possible. Inclusion criteria comprised: (1) any resistance training or majority resistant training-based study ≥ 4 weeks; (2) healthy trained or untrained participants with a mean age between 14 and 60 years; (3) training group with a minimum of four participants; (4) pre- and post-training means and standard deviations; and (5) sufficient information provided to quantify training intensity, volume, and frequency. Studies did not require a control or comparator group to be included. A standardised extraction codebook was developed using Microsoft Excel, with data extracted and coded independently by four researchers in duplicate, with one reviewer (AM) completing extraction for all studies to provide consistency. Study selection followed a two-stage selection strategy (title or abstract screen and full-text screen) undertaken primarily by AM and two researchers as part of the original larger review during 2018–2020. The independent screeners convened at the end of each screening stage to resolve any discrepancies. Data regarding the study (authors, year, total number of active intervention groups); participant characteristics (final study n, sex, training status, and age); outcome domain (maximum strength, power, jump performance, and sprinting performance); training dose (overall intensity, intensity of load, volume, frequency, number of exercises, number of sets, number of repetitions), and pre- and post-training means and standard deviations were obtained. The definitions used to categorise outcome domains included: (1) maximum strength: a measure of maximum force production where time was not limited (e.g., 1–6 repetition maximum, isometric mid-thigh pull, peak torque); (2) power: a direct measurement (e.g., Wingate test) or indirect estimation (e.g., vertical jump) of mechanical power output measured in Watts (absolute and normalised relative to body mass); (3) jump performance: measure of jump height or distance; (4) sprint performance: a measurement of the time to complete a specified linear distance or the velocity achieved; and (5) COD performance: a measurement of the time to complete a non-reactive change of direction or reactive task. Training status was categorised based on the mean S&C training experience as untrained (< 1 year), recreationally trained (1–5 years), or highly trained (> 5 years). If the mean S&C training experience was not stated, the minimum required experience was used for categorisation. Sex of the groups was categorised as male-only, female-only or mixed sex. Criteria used to quantify overall intensity and volume for each training mode can be found in Table 1. In brief, overall intensity was categorised specific to each domain and considered both intensity of effort and mechanical factors. In addition, intensity of load was also quantified based on the mean percentage of one repetition maximum (% 1RM) used in the resistance training. In cases where % 1RM was not explicitly stated, % 1RM values were estimated based on the number of repetitions performed per set using methods outlined by Haff and Triplett [20] (OSM 2). Training frequency was classified as the average number of sessions per week throughout the intervention.

**Table 1:** Criteria used to categorise overall intensity and volume of each training mode included

Training mode	Intensity categorisation	Volume categorisation
Resistance training	Coding based on % 1RM 1 = Low 0–59.9% 1RM 2 = Moderate 60–84.9% 1RM 3 = High ≥ 85% 1RM In cases where % 1RM was not explicitly stated, % 1RM value and category were estimated based on the number of repetitions performed per set using methods outlined by Haff and Triplett [19] (Online Supplementary Material 2)	Average number of repetitions performed per set in key exercises 1: Low – 1–5 2: Mid – 6–10 3: High – 11 +
Plyometric	Based on the exercises included, for example: 1 = Low – low amplitude hopping, box jumps, squat jumps 2 = Moderate – bounding, lateral jumps, hurdle jumps, countermovement jump, drop jump with < 30 cm drop 3 = High – drop jump with > 30 cm drop, multidirectional bounding, single leg jumps, rebounding jumps	Average number of foot contacts per session 1: Low – < 80 2: Mid – 80–120 3: High – 120 +
Ballistic	Always categorised as high intensity due to high levels of relative effort required, unless it was explicitly stated sub-maximal effort was used	Average number of repetitions performed per set 1: Low – 1–3 2: Mid – 4–6 3: High – 7 +
Sprint	Always categorised as high intensity due to high levels of relative effort required, unless it was explicitly stated sub-maximal effort was used (e.g. skipping, marching sub-maximal runs)	Average number of runs per session 1: Low – 1–4 2: Mid – 5–9 3: High 10 +
Change of direction	Based on exercises included: 1 = Low – ladder drills, footwork drills, single turn run with < 90^0^ change of direction (COD), 2 = Moderate – lateral movement drills, single turn with > 90^0^ COD, 3 = High – multiple sharp CODs, 505 drills, reactive drills	Average number of runs per session 1: Low – 1–5 2: Mid – 6–9 3: High 10 +
Combined	Combinations of resistance, sprint, ballistic, plyometric and agility training. To be considered a combined training mode, the secondary mode must account for at least 30% of total lower body training volume Categorisation of 1 = Low, 2 = Mid, 3 = High, based on categorisation of included training types	Categorisation of 1 = Low 2 = Mid 3 = High based on categorisation of included training types

*COD* change of direction, *cm* centimetre, *% 1RM* percentage one-repetition maximum

### Statistical Analysis

Effect sizes and their sampling variance were calculated using group mean and standard deviation values calculated pre-intervention and at any subsequent time-point. SMD_pre_ was calculated by dividing the relevant mean difference by the pre-intervention standard deviation. The sampling variance ${\sigma }_{e}^{2}({{\text{SMD}}}_{{\text{pre}}})$ of the effect size [21] was calculated using the following formula:

$${\sigma }_{e}^{2}\left({{\text{SMD}}}_{{\text{pre}}}\right)=\frac{n-1}{n\left(n-3\right)}\left(2\left(1-r\right)+n{{\text{SMD}}}_{{\text{pre}}}^{2}\right)-\frac{{{\text{SMD}}}_{{\text{pre}}}^{2}}{c{\left(n-1\right)}^{2}}$$

where $n$ is the sample size, $r$ is the correlation between repeated measures, and $c\left(n-1\right)$ is the bias function, which was approximated by $1-\frac{3}{4n-5}$[22]. To account for the small sample sizes generally used in S&C, a bias correction was applied to the effect size and sampling variance by multiplying by the approximated bias function and its square, respectively.

All meta-analyses were conducted using a nested four-level mixed-effects meta-analytic model. The series of nestings and a full overview of the model framework are presented in the Online Supplementary files (OSM 3). Predictors were added at level 2 (time of measurement from baseline), level 3 (outcome domain as a categorical predictor (strength, power, sprint, vertical jump, COD)) and level 4 (number of repetitions per set as a categorical predictor (low < 8, high ≥ 8); number of repetitions per set as a smooth predictor; number of sets; number of sets as a smooth predictor; number of sets as a categorical predictor (low < 4, high ≥ 4); number of sessions per week as a categorical predictor (low < 3, high ≥ 3); number of sessions per week as a smooth predictor; number of exercises as a categorical predictor (low < 4, high ≥ 4); number of exercises as a smooth predictor; volume as a categorical predictor (low < 2, mid = 2, high > 2); overall intensity as a categorical predictor (low < 2, mid = 2, high > 2); intensity of load (% 1RM) as a categorical predictor (low < 80; high ≥ 80); intensity of load (% 1RM) as a smooth predictor; sex as a categorical variable (males, females, mixed); and training status as a categorical predictor (untrained, recreationally trained, highly trained)).

Candidate models were fitted and compared based on predictive accuracy using ELPD-LOO (see OSM for further details) [23]. Median values and 95% credible intervals (CrIs) were presented for regression coefficients where predictors were found to improve the previous model, with the marginal effect of smooth terms visualised using plots and illustrating uncertainties. All analyses were conducted in R [24], with models fit using the brms package interfaced with Stan [25] to perform sampling, and leave-one-out cross-validation performed using the loo package [26]. Analyses were completed across the entire data set including both resistance-only and resistance-dominant training interventions, with a sensitivity analysis completed with resistance-only training interventions. Outlier SMD_pre_ values were identified by adjusting the distribution by a Tukey $g$-and-$h$ distribution and obtaining the 0.0035- and 0.9965-quantiles, with values beyond these points removed prior to further analysis [27]. Convergence of parameter estimates was obtained for all models with Gelman-Rubin R-hat values below 1.1 [28]. No attempts were made to assess certainty in the body of evidence for an outcome.

## Results

### Descriptions of Data

**Fig. 1:**
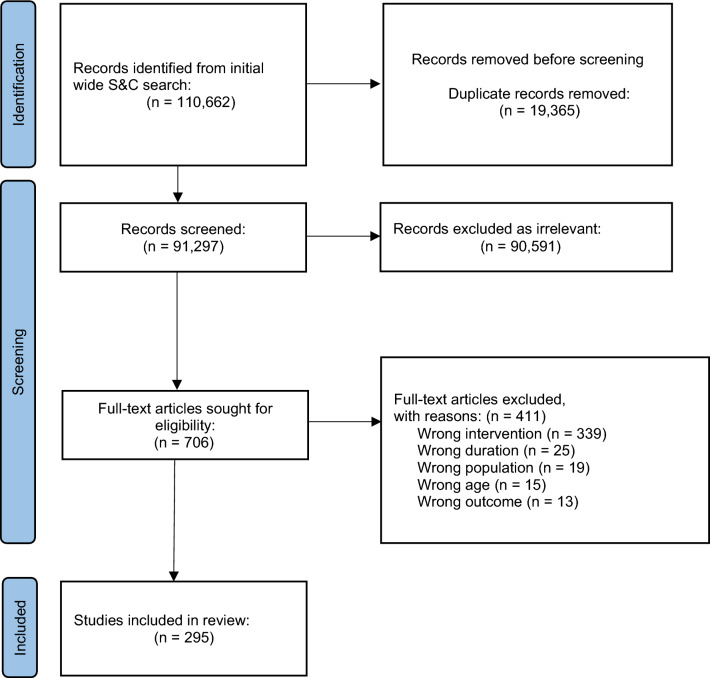
Preferred Reporting Items for Systematic Reviews and Meta-Analyses (PRISMA) flow diagram for included studies. *S&C* strength and conditioning

Data from a total of 295 studies comprising 535 groups and 6,710 participants were obtained (Fig. 1 and reference list provided in OSM 4). Of the 535 groups, 372 comprised resistance training only (n = 4,664), and 163 comprised resistance training combined with other training modalities (n = 2,046). Sixty-five percent of groups were categorised as male-only, 23% were categorised as mixed sex, and 12% were categorised as female-only. Fifty-four percent of groups were categorised as untrained, 41% were categorised as recreationally trained, and 5% were categorised as highly trained. The duration of interventions ranged from 4 to 208 weeks, with 97% of the data obtained from interventions ≤ 26 weeks. Analyses were thus restricted to time points ≤ 26 weeks following baseline, which provided data from 3,065 outcomes (maximum strength: 1,546 (50%); power: 550 (18%); jump performance: 512 (17%); sprint performance: 370 (12%); COD performance 87 (3%)). Results presented in text are from the complete data set comprising both resistance-only and resistance-dominant training interventions. Sensitivity analyses conducted with resistance-only training interventions were consistent with the larger data set. Full details of the best predictor models at each level for both resistance-only and resistance-dominant training interventions are presented in Tables 2 and 3.

**Table 2:** Best performing models at each stage of analysis for complete data set comprising both resistance-only and resistance-dominant training interventions

Level	Included data	Additional included variables [95% CrI]	ELPD-LOO [Standard error]	ELPD-LOO difference	Level 2 standard deviation [75% CrI]	Level 3 standard deviation [75% CrI]	Level 4 standard deviation [75% CrI]
1	291 studies 3004 outcomes	Null model; Mean: 0.55 [0.51–0.60]	– 1805 [60.9]		0.33 [0.31–0.36]	0.25 [0.24–0.26]	0.05 [0.02–0.07]
2	291 studies 3004 outcomes	Time (Weeks): 0.03 [0.02–0.04]	– 1792 [61.2]	– 12.1 [4.2]	0.33 [0.32–0.35]	0.24 [0.22–0.25]	0.05 [0.02–0.08]
2 + 3	291 studies 3004 outcomes	Outcome domain: Strength: 0.79 [0.74–0.84] Jump: 0.53 [0.47–0.59] Power: 0.52 [0.45–0.58] COD: 0.51 [0.39–0.63] Sprint: 0.39 [0.32–0.46]	– 1728 [62.0]	– 46.8 [12.0]	0.33 [0.31–0.36]	0.20 [0.19–0.22]	0.05 [0.02–0.08]
2 + 3 + 4	265 studies 2795 outcomes	Average number sets 0.05 [0.03–0.07] Smooth(Intensity value % 1RM)	– 1600 [59.9]	– 12.8 [3.8]	0.33 [0.31–0.36]	0.21 [0.20–0.22]	0.04 [0.01–0.07]
2 + 3*4	265 studies 2795 outcomes	Interaction between Outcome domain and Smooth(Intensity value % 1RM)	– 1588 [59.8]	– 11.7 [4.8]	0.33 [0.31–0.36]	0.20 [0.19–0.22]	0.05 [0.02–0.08]

*COD* change of direction, *CrI* credible interval

Table shows the additional variables included at each stage of the sequential process along with posterior estimates of model parameters, except for variables where smooth terms were added. At each stage, models also include variables identified as providing the best performance at the preceding stage

**Table 3:** Best performing models at each stage of analysis for resistance-only training interventions

Level	Included data	Additional Included variables [95% CrI]	ELPD-LOO [Standard error]	ELPD-LOO difference	Level 2 standard deviation [75% CrI]	Level 3 standard deviation [75% CrI]	Level 4 standard deviation [75% CrI]
1	197 studies 1,876 outcomes	Null model; Mean: 0.58 [0.52–0.64]	– 1197 [45.8]		0.36 [0.33–0.39]	0.26 [0.24–0.27]	0.10 [0.08–0.13]
2	197 studies 1,876 outcomes	Time (Weeks): 0.03 [0.02–0.04]	– 1189 [45.7]	– 8.3 [3.3]	0.36 [0.34–0.39]	0.24 [0.22–0.26]	0.11 [0.08–0.13]
2 + 3	197 studies 1,876 outcomes	Outcome domain: Strength: 0.79 [0.74–0.85] Jump: 0.54 [0.46–0.62] Power: 0.50 [0.42–0.58] COD: 0.58 [0.42–0.70] Sprint: 0.45 [0.36–0.55]	– 1162 [62.0]	– 18.4 [6.5]	0.34 [0.32–0.38]	0.22 [0.20–0.23]	0.10 [0.08–0.13]
2 + 3 + 4	177 studies 1,714 outcomes	Average number sets: 0.07 [0.04–0.09] Smooth(Intensity value %1RM)	– 1077 [41.6]	– 8.5 [3.1]	0.36 [0.33–0.40]	0.22 [0.20–0.24]	0.11 [0.09–0.13]
2 + 3*4	177 studies 1,714 outcomes	Interaction between Outcome domain and Smooth(Intensity value %1RM)	– 1061 [41.8]	– 12.0 [4.2]	0.35 [0.31–0.39]	0.23 [0.20–0.25]	0.10 [0.08–0.13]

*CrI* credible interval, *ELPD-LOO* expected log pointwise predictive density for a new dataset that was estimated with leave-one-out cross validation, *COD* change of direction

Table shows the additional variables included at each stage of the sequential process along with posterior estimates of model parameters, except for variables where smooth terms were added. At each stage, models also include variables identified as providing the best performance at the preceding stage

### Null Model

A total of 61 outliers were removed from the analysis such that effect sizes ranged from – 0.83 to 4.7. For the null model the pooled mean effect size was ${{\text{SMD}}}_{0.5}$ = 0.55 [95% CrI 0.51–0.60], with between-study standard deviation ${\tau }_{0.5}$= 0.33 [75% CrI 0.31–0.36] (Table 2).

### Level 2 Predictors

The only level 2 predictor included was time of measurement following baseline. The median time of measurement was 8 weeks (interquartile range (IQR): 6–12), with binary (short: ≤ 8 weeks, long: > 8 weeks) and linear predictors investigated. Binary categorisation showed an increase in mean pooled effect size with longer interventions (${{\text{SMD}}}_{{\text{short}}:{\text{long}},0.5}$= 0.17 [95% CrI 0.11–0.23]), and for the continuous linear predictor the weekly increase was estimated as ${{\text{SMD}}}_{{\upbeta }_{{\text{Time}},0.5}}$= 0.03 [95% CrI 0.02–0.04]. The ELPD-LOO difference comparing the null model and inclusion of categorical time or continuous time showed improved model performance and was equal to – 11.9 (se: 5.2) and – 12.1 (se: 4.2), respectively. All subsequent models including those assessed as part of the sensitivity analysis featured time as a linear predictor (Tables 2 and 3).

### Level 3 Predictors

**Fig. 2:**
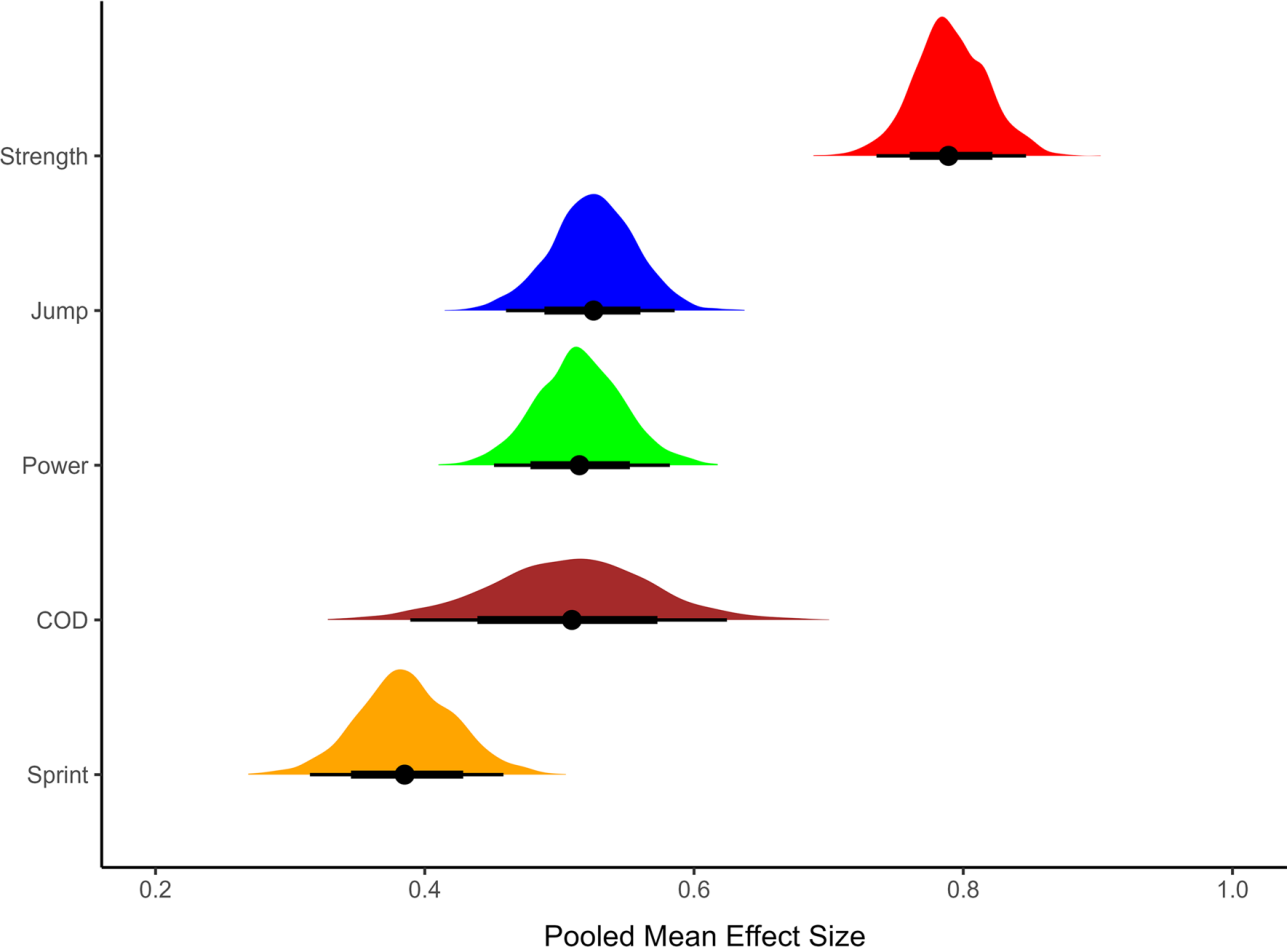
(Top): Posterior distributions of pooled mean effect sizes across outcome domains. (Bottom): Values represent shrunken values after fitting meta-analytic model also accounting for time of measurement. Black lines represent 75% and 95% credible intervals

The only level 3 predictor assessed was the outcome domain measured (Fig. 2). Using maximum strength as the reference level, the mean pooled value was higher in this domain compared to all others (${{\text{SMD}}}_{{\text{strength}}:{\text{COD}},0.5}$= – 0.28 [95% CrI – 0.39 to – 0.17]; ${{\text{SMD}}}_{{\text{strength}}:{\text{jump}},0.5}$= – 0.26 [95% CrI – 0.32 to – 0.21]; ${{\text{SMD}}}_{{\text{strength}}:{\text{power}},0.5}$ = – 0.28 [95% CrI – 0.33 to – 0.23]; ${{\text{SMD}}}_{{\text{strength}}:{\text{sprint}},0.5}$ = – 0.40 [95%CrI – 0.47 to – 0.34]). The effect of time remained positive after including outcome domain (${{\text{SMD}}}_{{\upbeta }_{{\text{Time}},0.5}}$= 0.03 [95%CrI 0.02–0.03]), with a large improvement in model performance (ELPD-LOO difference: – 46.8 (se: 12.0)). All subsequent models including those assessed as part of the sensitivity analysis featured time as a continuous predictor and outcome domain (Table 2 and 3).

### Level 4 Predictors

Initial analyses were conducted using the categorical volume and overall intensity predictors. No improvement in model performance relative to inclusion of time and outcome domain was obtained for volume (ELPD-LOO difference: + 5.2 (se: 4.6)). In contrast, improvement was observed for overall intensity (ELPD-LOO difference: – 6.6 (se: 2.2)) with evidence of greater mean pooled values for medium (${{\text{SMD}}}_{{\text{low}}:{\text{medium}},0.5}$= 0.07 [95% CrI 0.00–0.15]) and high categories (${{\text{SMD}}}_{{\text{low}}:{\text{high}},0.5}$= 0.10 [95% CrI 0.01–0.19]). The inclusion of smooth terms showed no improvement in model performance when adding the number of sessions per week (ELPD-LOO difference: + 3.4 (se: 2.0)) or the average number of exercises per session (ELPD-LOO difference: + 1.0 (se: 2.6)). Limited evidence of model improvement was obtained with inclusion of smooth terms for the average number of repetitions per session (ELPD-LOO difference: – 6.4 (se: 3.7)). The marginal effect illustrated a consistent decrease in effect size with a greater number of repetitions that slowed after ten repetitions. Stronger evidence of model improvement using smooth terms was obtained for intensity of load with values expressed as percentage of maximum (ELPD-LOO difference: – 18.0 (se: 4.5)) and the average number of sets per session (ELPD-LOO difference: – 17.5 (se: 4.4)) Marginal smooths illustrated that the relationship was monotonic but non-linear for intensity of load with a reduced incline between 65 and 100% of maximum. The relationship was found to be linear for the average number of sets per session, with an increasing mean effect size association with a greater number of sets (${{\text{SMD}}}_{{\upbeta }_{{\text{Sets}},0.5}}$ = 0.05 [95% CrI 0.03–0.07]).

No improvement in model performance was obtained for the addition of sex (ELPD-LOO difference: + 2.9 (se: 2.3)) or training status (ELPD-LOO difference: + 0.2 (se: 2.3)). The same lack of improvement in model performance was obtained with sensitivity analyses conducted with resistance-only interventions. A final best performing model including both the average number of sets per session (${{\text{SMD}}}_{{\upbeta }_{{\text{Sets}},0.5}}$ = 0.05 [95% CrI 0.03–0.07]) and smoothed intensity of load was obtained for the complete data set and for resistance-only training interventions (Table 2 and 3).

### Cross-Level Interactions Between Levels 3 and 4

**Fig. 3:**
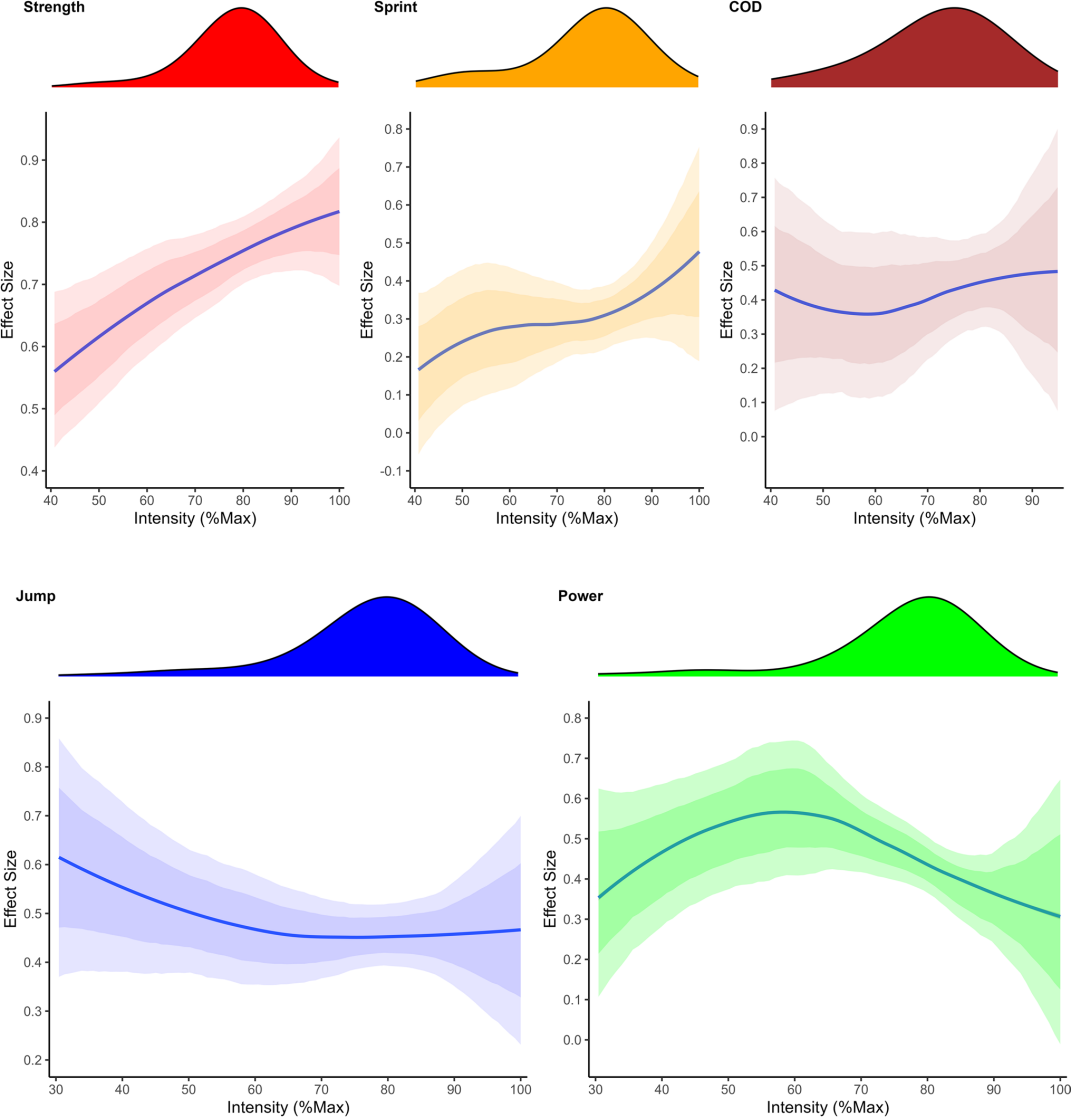
(Top): Marginal effects of smooth terms illustrating interactions between intensity of load expressed as percentage of maximum and outcome domain. (Bottom): Density plots at the top of each figure illustrate the distribution of the load intensity variable for the given outcome domain. Solid lines represent the best estimate of the smooth relationship and shaded regions represent intervals of uncertainty (75% and 95%)

Potential cross-level interactions were investigated separately between outcome domain and both average number sets per session and intensity of load. No improvement in model performance was obtained for the cross-level interaction between outcome domain and average number of sets per session (ELPD-LOO difference: – 0.6 (se: 3.5)). In contrast, model performance was increased for the cross-level interaction between outcome domain and intensity of load (ELPD-LOO difference: – 0.6 (se: 3.5)), with spline modelled intensity showing markedly different relationships across the different outcome domains (Table 2 and Fig. 3).

## Discussion

The primary aim of this meta-analysis was to produce a comprehensive modelling of the dose–response relationships between resistance exercise and commonly used measures of physical performance within S&C. The analyses identified that a range of factors are associated with the magnitude of change across resistance-only and resistance-dominant training interventions. These factors include the length of the intervention, outcome type, volume, overall intensity of training, and the intensity of load. The analyses also identified important interactions between loading intensity and outcome domain, such that some outcomes are more likely to experience greater improvements with a range of sub-maximum loads (30–70% 1RM).

Length of intervention has generally not been explored in dose–response modelling due to most reviews focusing on a smaller number of homogeneous studies with a restricted range of durations. Across the studies included in this meta-analysis, durations were found to be relatively short, with the median duration equal to 8 weeks and 97% of interventions lasting less than 26 weeks. The prevalence of shorter duration studies may be due to multiple challenges associated with longer duration interventions including an increased need for resources as well as greater inclusion and agreement from key stakeholders (athlete, organisation and management), increased dropouts, and scheduling difficulties to fit within non-competitive periods [29]. Despite the relatively short intervention durations, the results show that substantive improvements can be made, and that longer durations within these time frames create greater mean improvements. Therefore, practitioners and athletes could use shorter time periods, potentially during off-season or preseason, to maximise physiological improvements when competition or sporting demands are lowest. The effect of duration remained consistent throughout the model-building process with standardised mean differences estimated to increase by approximately 0.03 for each additional week of training. Given the relatively short and homogeneous durations included, there was limited ability to explore the functional form of changes over longer durations (e.g., playing seasons, years). In a recent large modelling study investigating the time-course of strength adaptations, Steele et al. [30] showed that linear-log growth models were appropriate to describe improvements of relatively untrained participants over the course of almost 7 years, with improvements tending to plateau after approximately 1 year. The training stimulus investigated by Steele et al. [30] focused on minimal dose resistance training (1x/week, single sets to momentary failure of six exercises), which is likely to have influenced the parameters obtained. Our analysis was limited to durations of no more than 26 weeks and thus we cannot draw inferences as to how results might change over longer time frames. Further research is required to better understand the influence of duration of an intervention and likely interactions between participant characteristics, the specific outcome, the training stimulus, and changes in the training stimulus following, for example, different periodized approaches.

The manipulation of acute program variables within resistance training interventions is often focused on the development of a single outcome domain. Previous meta-analyses investigating dose–response relationships have predominantly focused on the development of muscular strength and/or hypertrophy [7–17]. The current meta-analysis demonstrated varying effects across multiple outcome domains commonly targeted with the greatest effect sizes obtained for strength, and the lowest obtained for sprint performance. Resistance training for the purpose of improving maximum strength is arguably the most investigated and well-understood area within S&C [1, 2], and greater effect sizes may reflect this increased refinement and specificity between traditional resistance-training methods and maximum strength outcomes [1]. In addition, researchers frequently test maximum strength using the same exercises included in the training intervention, further increasing specificity and potentially the improvements measured [31]. Results from a previous meta-analysis indicate that the dose–response relationship between the % 1RM and strength gains diminishes when testing is carried out isometrically [15]. Practitioners should be advised to select an appropriate testing mode for the given training stimulus applied, whilst being aware of any potential upward or downward shift in expected results dependent on the measurement used. Further study is needed to provide greater context to the transfer of strength from varied magnitudes of load to neutral testing modalities.

Following maximum strength, jump performance and power generated the next largest effects. Similar magnitude improvements in jump performance and power may be expected given the well-established relationship between the two factors [32–35]. In addition, many of the studies measured power during loaded and unloaded jumps, further increasing associations and similar magnitude improvements. Outcomes relating to sprint performance demonstrated the lowest magnitude improvements. Sprinting comprises a substantial and complex technique element [36–40], and given the relatively low number of studies (~ 12%) that included sprint specific interventions, a lower effect size distribution may be expected. More broadly, the lower effect size distribution for sprint outcomes may also reflect a lack of specificity with regards to development of relevant physical outputs. Transference between improvements and long-term adaptations in S&C is dependent primarily on the training principles of specificity and progressive overload, respectively [41, 42]. Most training methods included in the meta-analysis focused on bilateral production of maximum vertical forces over long durations. In contrast, sprinting activities require high forces produced over short ground contact times that are predominantly unilateral with substantive horizontal components in relation to the body’s position relative to the ground [37, 38, 43, 44]. In a recent meta-analysis, Murphy et al. [4] showed moderate to strong relationships between improvements in strength, power and sprint performance in team sport athletes, concluding that greater development of physical capacities may result in further improvements in sprint performance. Despite these correlations, however, researchers have also shown that large increases in maximum strength (~ 12–18%) translate into only small decreases in sprint times (~ -2–8%) [45–47]. With evidence to suggest restrictions may exist in the transference of physical capacities to sprinting, further sprint-specific training modes such as resisted sprint training may provide additional benefits allowing for the ability to overload kinetic output with increased kinematic specificity to the complex movement of sprinting [48]. Collectively, there appears to be scope for future research to investigate why improvements in sprint performance are generally much smaller than other outcome domains and whether this difference can be ameliorated with a focus on certain training practices.

Movements associated with COD and agility could be considered even more complex than sprinting due to the high acceleration and deceleration demands, the ability to rapidly alter body position, combined with the need in some activities to react to an external stimulus [49, 50]. The results of the current meta-analysis suggest improvements in COD are likely to be of similar magnitude to those measured during vertical jump and tasks focusing on the development of power, albeit with a greater level of uncertainty. Outcomes measuring COD and the related construct of change of direction speed and agility represent a developing area within S&C [51, 52] with only ~ 3% of outcomes assessing COD performance. Whilst reasons for the larger effect size distribution in comparison to linear sprinting require further study, potential explanations include the complex, multifaceted nature of the tasks and the scope for multiple limiting factors to be addressed. Additionally, it is recognised that many agility and COD tasks include substantive skill elements [52], such that failure to appropriately familiarize participants could lead to systematic biases in regard to learning effects and subsequent overestimations of effect sizes.

The results of the current meta-analysis demonstrate the importance of training intensity. Previous researchers investigating dose–response relationships have tended to contextualise and quantify intensity based on load and thereby % 1RM [7, 8, 14, 15]. This approach works best when considering traditional strength or hypertrophy focused interventions comprising large compound movements where 1RMs can be measured and appropriately summarise a relevant feature of intensity [53]. An aim of the current meta-analysis was to investigate dose–response relationships across a range of resistance-based training modalities and outcomes; therefore, in addition to intensity of load expressed as a % 1RM, a more general categorisation scheme was included. Interventions comprising predominantly ballistic, loaded jumping or sprinting exercises were always considered high intensity, due to the high mechanical loads and assumption that they are conducted with maximal intent, unless stated otherwise. Across all outcomes, evidence was obtained that greater overall intensity was associated with increased effect sizes, with interventions judged to be of medium and high overall intensity expected to increase effect sizes by approximately 0.10 relative to low overall intensity. When prescribing intensity, practitioners should consider training intensity beyond simply intensity of load (% 1RM). For example, ensuring maximum intent during ballistic exercise exemplifies a simplistic method of prescribing higher intensity training. Incorporating methods such as velocity-based training provides the opportunity for instantaneous feedback during ballistic movements to encourage maximal intent [54]. Adjusting the intensity of plyometric training may be more complex and largely dependent on exercise selection to increase or decrease take-off and landing ground reaction forces [55].

Additional detailed information on the dose–response relationship of intensity was obtained when investigating potential interaction effects between outcome and intensity of load measured by % 1RM. The results identified a range of different profiles, with no clear pattern for COD, monotonic increases for strength and speed, a monotonic decrease for jump performance, and a parabolic profile for power. The best estimate profile for maximum strength appeared non-linear with an inflection point ~ 70% 1RM, where further increases in effect size estimates started to slow with additional load. The results of the present meta-analysis are consistent with previous reviews, e.g. Peterson et al. [8] identified increased effects with heavier % 1RM loads but diminishing effects, particularly with untrained participants [18]. The results also align with previous research indicating that heavy load training may increase muscle activation by up to 30% [56], conceivably providing a stronger stimulus for adaptation. Some authors have also suggested, however, that improvements in strength with greater loads may be inflated due to high specificity of task and outcome, given that strength testing is often conducted performing the 1RM of the movement being trained [15].

Analysis of sprint performance also identified a monotonic increase in effect, with the greatest increases obtained with the heaviest loads. The most common sprint outcomes investigated in S&C research include the time to sprint between 5 and 50 m, with the most frequent intervals comprising 10, 20 and 30 m [4]. Most studies have been conducted with either team sport or untrained participants who achieve maximum velocity between 15 and 40 m, in comparison to trained sprinters who require distances of 40–80 m to achieve maximum velocity [38, 44, 57, 58]. Consequently, sprint data collected over 10–30 m may provide researchers with divergent outcomes describing both acceleration and maximum velocity. Previous studies have reported strong associations with outcomes designed to assess acceleration (e.g., 10 m) and horizontal force, power and relative strength with longer duration ground contact times (approximately 200 ms) [37, 38]. In contrast, maximum velocity sprinting has been shown to be dependent on the ability to maintain large horizontal and vertical forces whilst minimizing braking forces with reduced ground contact time (approximately 100 ms) [38, 39, 58]. Previous meta-analyses have concluded that high-intensity non-specific resistance exercise is among the most effective training methods to improve sprint performance in team sport athletes [4, 36, 59]. Neural and morphological adaptations associated with high-load resistance exercise and improved force output may provide a mechanism for positive transfer to the high levels of horizontal force required during early-phase acceleration to improve sprint performance. Highly trained individuals may require more specific training methods, however, that target improvements in physical qualities while matching the kinematic demands of sprinting [1, 4].

In contrast to the increasing dose–response relationships with intensity for strength and sprint performance, results identified monotonic decreases for jump performance with the largest effects obtained at ~ 30% 1RM. Jump performance is dependent on net impulse and take-off velocity such that % 1RM loads lifted with maximum intent provide sufficient stimulus but do not limit velocity to a large extent may provide the greatest transfer to improvements in jump performance [33, 60]. Previous researchers have also demonstrated that jump squat training with low (< 30% 1RM) or no additional load can produce velocity-specific adaptations associated with improvements in jump performance [61]. The intensity profile for outcomes measuring power production was parabolic, with the greatest improvements obtained between ~ 40 and 70% 1RM. These results support the hypothesis that performing resistance exercise with loads that elicit the largest power outputs is the most effective method to improve power and that for most exercises power is maximised between 30 and 70% 1RM [62]. During weightlifting exercises (clean, snatch, hang and pull variations), power is maximised with heavier loads (≥ 70% 1RM), whereas loads of 0–30% 1RM maximise power during jump squat exercises [62]. As the optimum load for power production is exercise-dependent, practitioners should be aware of the appropriate load required to stimulate peak power output within the prescribed exercises and endeavour to create athlete-specific profiles where access to relevant measurement devices is available.

The influence of training frequency and volume on strength and hypertrophy has been assessed in several previous meta-analyses [10–12, 16]. The results obtained herein were mixed but showed limited evidence that these factors were influential. No improvement in model performance was obtained when including the number of sessions per week as a categorical or continuous predictor, or the average number of exercises per session. Only limited evidence of model improvement was obtained for the average number of repetitions per set, with the marginal effect showing declines as the number of repetitions increased. In contrast, evidence was obtained for greater effect sizes with increasing number of sets per session. Seminal research by Rhea et al. [9] was among the first, within S&C, to use meta-analytical techniques to assess the use of single versus multiple sets in resistance exercise for strength development. The authors concluded multiple sets were more beneficial than single-set training. A follow-up meta-regression by Krieger et al. [63] found a 46% increase in muscular strength when completing two to three sets, in comparison to single sets, although no further difference was found for resistance exercise with more than four sets. More recently, researchers have concluded that increasing weekly training volume through increased number of sets can produce similar results to increasing training frequency [11, 12]. The current meta-analysis demonstrates that focussing on a smaller number of key exercises while completing multiple sets at an appropriate intensity for a targeted outcome may be more beneficial than attempting to perform many exercises with an increased frequency.

The training status of participants is a key consideration when designing and implementing resistance exercise. Previous meta-analyses have demonstrated rank-order effects, with the largest improvements obtained by untrained participants [1, 7], followed by recreationally trained and then highly trained participants. In contrast, the current meta-analysis found a lack of evidence to support different effects across the training status categories. Differences in results obtained in the present versus previous meta-analyses may be due to several reasons. Previous analyses have been less formal than those conducted herein, with authors identifying differences based primarily on point estimates. In contrast, participant training status was assessed in the present analysis with predictor variables for lower levels already included in the model and addition of the factor was assessed based on ability to improve model performance. The lack of data for highly trained participants, disproportionate inclusion of untrained participants, combined with short-duration (≤ 26 weeks) interventions, which are known limitations within S&C research [64], may have also influenced the results obtained and discordance with what is generally believed in the field. With advancements in technology and ability to collect valid and reliable high-frequency data over longer periods across all levels of sport and recreation, use of longitudinal data collected in the field provides opportunities to better investigate differences in dose-responses relative to participant training status.

In addition to training status, the current meta-analysis found a lack of evidence to support different effects between sexes. Although males exhibit greater levels of baseline strength and muscle mass [65], the current meta-analysis results are consistent with previous research that has failed to identify any difference in effects between sexes in improvements in strength or hypertrophy, with indications that varying levels of adaptations may be more related to relative strength [10, 65–67]. A previous meta-analysis conducted by de Villareal et al. [68], however, reported greater improvement in males following plyometric training relative to females. The authors were unable to provide a strong rationale for the finding, and speculated that large differences in sample sizes between the sexes may have confounded results. Research has shown that stronger individuals are able to produce a greater rate of force development and power during time-restricted tasks [3], and so there is the potential that increased strength at baseline may be advantageous for plyometric training. Further research is required to identify potential differences in the dose–response relationship between sexes and more complex sport-specific outcomes. Female participants are largely under-represented in S&C research, with only 12% of the studies included here conducted with female-only groups. In addition, research suggests that only 39% of all published sport science data are collected with female participants [69]. To better address the question of potential differences in dose–response relationships, more female participant data are required.

Whilst this is the most comprehensive meta-analysis to date investigating dose–response relationships between resistance and resistance-dominant interventions, there are multiple limitations that should be considered when interpreting the findings. There are clear limitations in summarising different dose components from a training intervention based on, for example, the average number of sets, where this variable may change substantially depending on the periodisation or progression model. In addition, for variables such as overall training volume and intensity, there was a high degree of subjectivity and challenge in obtaining a single value, particularly when considering different training modes. The current meta-analysis was intended to uncover general relationships, but the extensive heterogeneity across the data set limits nuance and there are limitations in drawing strong inferences from pooling of indirect data. It is expected that there will be many instances where factors such as training frequency and volume strongly influence the effectiveness of an intervention; however, variables quantifying frequency and volume in the present analysis lacked predictive power across this large and heterogeneous data set. Despite these results and considering the limitations of the meta-analysis, practitioners are still recommended to implement periodized training interventions that include appropriate manipulations in intensity, volume and frequency over time in an attempt to maximise a given outcome. Overall, the results of the present meta-analysis suggest that practitioners should focus first on overall intensity and intensity of load with the appropriate target outcomes in mind.

## Conclusion

The current meta-analysis is the most comprehensive to date to investigate dose–response relationships of resistance and resistance-dominant training with respect to a range of commonly studied outcome domains in S&C research. The findings are that resistance exercise is effective over relatively short durations (~ 8 weeks) and extending a single intervention over longer periods is likely to result in further improvements. The expected magnitude of improvement appears to be predominantly influenced by intensity and the outcome domain measured. Performance of resistance training with a higher intensity as measured by a composite of the effort applied, the difficulty of the exercise, and maximising target biomechanical quantities result in greater improvements. When considering the magnitude of the load lifted as a % 1RM, the profile that creates the greatest improvements is dependent on the outcome domain. Improvements in strength are likely to be maximised with the heaviest loads, whereas vertical jump performance may be maximised with relatively light loads (~ 30% 1RM), and power with low to moderate loads (40–70% 1RM). Sprinting performance represents the most difficult outcome domain to improve with resistance and resistance-dominant training, and this may be influenced by lower specificity.

### Supplementary Information

Below is the link to the electronic supplementary material.

Supplementary file1 (DOCX 64 KB)
