# Supplementary material for: Dose–Response Modelling of Resistance Exercise Across Outcome Domains in Strength and Conditioning: A Meta-analysis
Source: Sports Med. 2024 Apr 23;54(6):1579–94. doi: 10.1007/s40279-024-02006-3 (PMC11239729; doi:10.1007/s40279-024-02006-3)
Supplement: Supplementary file 1 — Supplementary file1 (DOCX 64 KB) [file 40279_2024_2006_MOESM1_ESM.docx]

**Supplementary File 1:** Checklist of Preferred Reporting items for Systematic Review and Meta-Analysis

| **Section and Topic** | **Item #** | **Checklist item** | **Location where item is reported** |
| --- | --- | --- | --- |
| **TITLE** | | |  |
| Title | 1 | Identify the report as a systematic review. | Reported as a meta-analysis. Search is not exhaustive and does not include all relevant research. |
| **ABSTRACT** | | |  |
| Abstract | 2 | See the PRISMA 2020 for Abstracts checklist. |  |
| **INTRODUCTION** | | |  |
| Rationale | 3 | Describe the rationale for the review in the context of existing knowledge. | 4-6 |
| Objectives | 4 | Provide an explicit statement of the objective(s) or question(s) the review addresses. | 6,7 |
| **METHODS** | | |  |
| Eligibility criteria | 5 | Specify the inclusion and exclusion criteria for the review and how studies were grouped for the syntheses. | 8 |
| Information sources | 6 | Specify all databases, registers, websites, organisations, reference lists and other sources searched or consulted to identify studies. Specify the date when each source was last searched or consulted. | 7.8 |
| Search strategy | 7 | Present the full search strategies for all databases, registers and websites, including any filters and limits used. | Not included. |
| Selection process | 8 | Specify the methods used to decide whether a study met the inclusion criteria of the review, including how many reviewers screened each record and each report retrieved, whether they worked independently, and if applicable, details of automation tools used in the process. | 8 |
| Data collection process | 9 | Specify the methods used to collect data from reports, including how many reviewers collected data from each report, whether they worked independently, any processes for obtaining or confirming data from study investigators, and if applicable, details of automation tools used in the process. | 8 |
| Data items | 10a | List and define all outcomes for which data were sought. Specify whether all results that were compatible with each outcome domain in each study were sought (e.g. for all measures, time points, analyses), and if not, the methods used to decide which results to collect. | 8.9 |
|  | 10b | List and define all other variables for which data were sought (e.g. participant and intervention characteristics, funding sources). Describe any assumptions made about any missing or unclear information. | 9 |
| Study risk of bias assessment | 11 | Specify the methods used to assess risk of bias in the included studies, including details of the tool(s) used, how many reviewers assessed each study and whether they worked independently, and if applicable, details of automation tools used in the process. | Stated that risk of bias was not assessed on pg 8 |
| Effect measures | 12 | Specify for each outcome the effect measure(s) (e.g. risk ratio, mean difference) used in the synthesis or presentation of results. | 11 |
| Synthesis methods | 13a | Describe the processes used to decide which studies were eligible for each synthesis (e.g. tabulating the study intervention characteristics and comparing against the planned groups for each synthesis (item #5)). | 11,12 |
|  | 13b | Describe any methods required to prepare the data for presentation or synthesis, such as handling of missing summary statistics, or data conversions. | 12 |
|  | 13c | Describe any methods used to tabulate or visually display results of individual studies and syntheses. | 12 |
|  | 13d | Describe any methods used to synthesize results and provide a rationale for the choice(s). If meta-analysis was performed, describe the model(s), method(s) to identify the presence and extent of statistical heterogeneity, and software package(s) used. | 12 |
|  | 13e | Describe any methods used to explore possible causes of heterogeneity among study results (e.g. subgroup analysis, meta-regression). | 13 |
|  | 13f | Describe any sensitivity analyses conducted to assess robustness of the synthesized results. | 13 |
| Reporting bias assessment | 14 | Describe any methods used to assess risk of bias due to missing results in a synthesis (arising from reporting biases). | Stated that risk of bias was not assessed on pg 8 |
| Certainty assessment | 15 | Describe any methods used to assess certainty (or confidence) in the body of evidence for an outcome. | Stated that no methods were used to assess certainty in the body of evidence for an outcome on pg 13 |
| **RESULTS** | | |  |
| Study selection | 16a | Describe the results of the search and selection process, from the number of records identified in the search to the number of studies included in the review, ideally using a flow diagram. | 15 |
|  | 16b | Cite studies that might appear to meet the inclusion criteria, but which were excluded, and explain why they were excluded. | 15 |
| Study characteristics | 17 | Cite each included study and present its characteristics. | Citations for included studies presented in supplementary 3. |
| Risk of bias in studies | 18 | Present assessments of risk of bias for each included study. | Stated that risk of bias was not assessed on pg 8 |
| Results of individual studies | 19 | For all outcomes, present, for each study: (a) summary statistics for each group (where appropriate) and (b) an effect estimate and its precision (e.g. confidence/credible interval), ideally using structured tables or plots. | 15-21 |
| Results of syntheses | 20a | For each synthesis, briefly summarise the characteristics and risk of bias among contributing studies. | 15-21 |
|  | 20b | Present results of all statistical syntheses conducted. If meta-analysis was done, present for each the summary estimate and its precision (e.g. confidence/credible interval) and measures of statistical heterogeneity. If comparing groups, describe the direction of the effect. | 15-21 |
|  | 20c | Present results of all investigations of possible causes of heterogeneity among study results. | 15-21 |
|  | 20d | Present results of all sensitivity analyses conducted to assess the robustness of the synthesized results. | 16-20 |
| Reporting biases | 21 | Present assessments of risk of bias due to missing results (arising from reporting biases) for each synthesis assessed. | Stated that risk of bias was not assessed on pg 8 |
| Certainty of evidence | 22 | Present assessments of certainty (or confidence) in the body of evidence for each outcome assessed. | Stated that no methods were used to assess certainty in the body of evidence for an outcome on pg 13 |
| **DISCUSSION** | | |  |
| Discussion | 23a | Provide a general interpretation of the results in the context of other evidence. | 22-30 |
|  | 23b | Discuss any limitations of the evidence included in the review. | 30 |
|  | 23c | Discuss any limitations of the review processes used. | 30 |
|  | 23d | Discuss implications of the results for practice, policy, and future research. | 31 |
| **OTHER INFORMATION** | | |  |
| Registration and protocol | 24a | Provide registration information for the review, including register name and registration number, or state that the review was not registered. | Stated that this is a follow-on review from a previous review. |
|  | 24b | Indicate where the review protocol can be accessed, or state that a protocol was not prepared. | No protocol was prepared. |
|  | 24c | Describe and explain any amendments to information provided at registration or in the protocol. | No protocol was prepared. |
| Support | 25 | Describe sources of financial or non-financial support for the review, and the role of the funders or sponsors in the review. | 31 |
| Competing interests | 26 | Declare any competing interests of review authors. | 31 |
| Availability of data, code and other materials | 27 | Report which of the following are publicly available and where they can be found: template data collection forms; data extracted from included studies; data used for all analyses; analytic code; any other materials used in the review. | Supplementary files include checklist, conversion chart, and included references. |

**Supplementary File 2:** Table outlining %1RM estimation based on repetitions performed, adapted from Haff and Triplett [19].

| Repetitions Performed | %1RM |
| --- | --- |
| 1 | 100 |
| 2 | 95 |
| 3 | 93 |
| 4 | 90 |
| 5 | 87 |
| 6 | 85 |
| 7 | 83 |
| 8 | 80 |
| 9 | 77 |
| 10 | 75 |
| 11 | 70 |
| 12 | 67 |
| 15 | 65 |

**Supplementary File 3:** Further details of statistical approach

All meta-analyses were conducted using a nested four-level mixed effects meta-analytic model. The series of nestings included the individual study (level 4), the outcome (level 3), the measurement occasion (level 2) as many studies included more than just pre- and post-intervention assessments, and the within study sampling variance (level 1). A representation of the meta-analyses conducted includes:

$$Level1: d_{ijk} =\beta_{0ijk}+e_{ijk}, e_{ijk}\sim N(0,\sigma_{e}^{2})$$

$$Level2: \beta_{0ijk}=\eta_{0jk}+\beta_{2,1}x_{2,1ijk}+r_{ijk}, r_{ijk}\sim N(0,\sigma_{r}^{2})$$

$Level3: \eta_{0jk} =\theta_{0k}+\beta_{3,1}x_{3,1jk}+\beta_{3,2}\left( x_{3,1jk}*x_{4,1k} \right)+u_{0jk},$ $u_{0jk}\sim N(0,\sigma_{u}^{2})$

$$Level4: \theta_{0k} =\gamma_{0}+\beta_{4,1}x_{4,1k}+s\left( x_{4,2k} \right)+v_{0k}, v_{0k}\sim N(0,\sigma_{v}^{2})$$

where $d_{ijk}$is the observed effect size at measurement occasion $i (i=1,2,\ldots,I_{jk})$, from outcome $j (j=1,2,\ldots,J_{k})$ and from study $k (k=1,2,\ldots,K)$. The indexing $I_{jk}$denotes that the number of measurement occasions may vary across outcomes and studies, and $J_{k}$denotes the number of outcomes may vary across studies. ​The random effects across the different levels ($v_{0k},u_{0jk},r_{ijk},e_{ijk}$) were assumed to be independent. $\beta$ terms represent regression coefficients for the predictor variables $x$ included at levels 2 to 4. Cross-level interactions are denoted by $*$ and for some continuous predictors, smooth functions (simple basis functions) were used to model non-linear effects of the predictor and are denoted by $s\left( x \right)$.

Candidate models were fitted and compared based on predictive accuracy using the theoretical expected log pointwise predictive density (ELPD) for a new dataset that was estimated with leave-one-out cross validation (ELPD-LOO) [23]. The ELPD-LOO generates a standard error that describes the uncertainty in the predictive performance for unknown future data. Candidate models were fit gradually increasing the number of predictors starting at level 2 and progressing to level 4. The ELPD-LOO difference between a new and previous model was calculated and addition of the predictor judged as an improvement and maintained in subsequent models if the value was at least two times the standard error.

**Supplementary File 4:** Reference list of included studies

1. Hoffman JR, Cooper J, Wendell M, Kang J. Comparison of Olympic vs. traditional power lifting training programs in football players. *Journal of Strength and Conditioning Research*. 2004; 18(1):129-35.
2. Balabinis CP, Psarakis CH, Moukas M, Vassiliou MP, Behrakis PK. Early phase changes by concurrent endurance and strength training. *Journal of Strength and Conditioning Research.* 2003; 17(2):393-401.
3. Carlson K, Magnusen M, Walters P. Effect of various training modalities on vertical jump. *Research in Sports Medicine.* 2009; 17(2):84-94.
4. Kyrolainen H, Avela J, McBride JM, Koskinen S, Andersen JL, Sipila S, et al. Effects of power training on muscle structure and neuromuscular performance. *Scandinavian Journal of Medicine and Science in Sports.* 2005; 15(1):58-64.
5. Whitehead MT, Scheett TP, McGuigan MR, Auckland NZ, Martin AV. A Comparison of the Effects of Short-Term Plyometric and Resistance Training on Lower Body Muscular Performance. *Journal of Strength and Conditioning Research.* 2018; 32(10):2743-2749
6. Zaras N, Spengos K, Methenitis S, Papadopoulos C, Karampatsos G, Georgiadis G, Stasinaki A, Manta P, Terzis G. Effects of strength vs. ballistic-power training on throwing performance. *Journal of sports science & medicine*. 2013; 12(1):130.
7. Argus CK, Gill ND, Keogh JWL, McGuigan MR, Hopkins WG. Effects of Two Contrast Training Programs on Jump Performance in Rugby Union Players During a Competition Phase. *International Journal of Sports Physiology & Performance*. 2012;7(1):68-75
8. Holmstrup ME, Jensen BT, Evans WS, Marshall EC. Eight Weeks of Kettlebell Swing Training Does not Improve Sprint Performance in Recreationally Active Females. *International Journal of Exercise Science.* 2016; 9(3):437-444.
9. Ronnestad BR, Kojedal O, Losnegard T, Kvamme B, Raastad T. Effect of heavy strength training on muscle thickness, strength, jump performance, and endurance performance in well-trained Nordic Combined athletes. *European journal of applied physiology.* 2012; 112(6):2341-2352.
10. Folland JP, Irish CS, Roberts JC, Tarr JE, Jones DA. Fatigue is not a necessary stimulus for strength gains during resistance training. *British journal of sports medicine*. 2002; 36(5):370-373.
11. Norrbrand L, Pozzo M, Tesch PA. Flywheel resistance training calls for greater eccentric muscle activation than weight training. *European journal of applied physiology.* 2010; 110(5):997-1005.
12. Sander A, Keiner M, Wirth K, Schmidtbleicher D. Influence of a 2-year strength training programme on power performance in elite youth soccer players. *European journal of sport science*. 2013; 13(5):445-51.
13. Gonzalez-Badillo JJ, Rodriguez-Rosell D, Sanchez-Medina L, Gorostiaga EM, Pareja-Blanco F. Maximal intended velocity training induces greater gains in bench press performance than deliberately slower half-velocity training. *European journal of sport science.* 2014; 14(8):772-781.
14. Cantrell GS, Schilling BK, Paquette MR, Murlasits Z. Maximal strength, power, and aerobic endurance adaptations to concurrent strength and sprint interval training. European journal of applied physiology. 2014; 114(4):763-71.
15. Sarabia JM, Fernandez-Fernandez J, Juan-Recio C, Hernández-Davó H, Urbán T, Moya M. Mechanical, hormonal and psychological effects of a non-failure short-term strength training program in young tennis players. *Journal of human kinetics*. 2015; 45:81-91.
16. Remaud A, Cornu C, Guevel A. Neuromuscular adaptations to 8-week strength training: Isotonic versus isokinetic mode. *European journal of applied physiology.* 2010; 108(1):59-69.
17. Harris GR, Stone MH, O'Bryant HS, Proulx CM, Johnson RL. Short-term performance effects of high power, high force, or combined weight-training methods. *Journal of Strength and Conditioning Research*. 2000; 14(1):14-20.
18. Brito J, Vasconcellos F, Oliveira J, Krustrup P, Rebelo A. Short-term performance effects of three different low-volume strength-training programmes in college male soccer players. *Journal of human kinetics*. 2014; 40:121-128.
19. Cholewa JM, Rossi FE, MacDonald C, Hewins A, Gallo S, Micenski A, et al. The effects of moderate- versus high-load resistance training on muscle growth, body composition, and performance in collegiate women. *Journal of Strength and Conditioning Research.* 2018; 32(6):1511-24.
20. James LP, Haff GG, Kelly VG, Connick M, Hoffman B, Beckman EM. The impact of strength level on adaptations to combined weightlifting, plyometric and ballistic training. *Scandinavian Journal of Medicine & Science in Sports.* 2018; 28(5):1494-505.
21. Coratella G, Milanese C, Schena F. Unilateral eccentric resistance training: A direct comparison between isokinetic and dynamic constant external resistance modalities. *European Journal of Sport Science*. 2015; 15(8):720-6.
22. Vantarakis A, Chatzinikolaou A, Avloniti A, Vezos N, Douroudos II, Draganidis D, Jamurtas AZ, Kambas A, Kalligeros S, Fatouros IG. A 2-month linear periodized resistance exercise training improved musculoskeletal fitness and specific conditioning of navy cadets. *Journal of Strength and Conditioning Research*. 2017; 31(5):1362-70.
23. Boyer, Brian T. A Comparison of the Effects of Three Strength Training Programs on Women. *Journal of Strength and Conditioning Research*. 1990; 4(3): 88-94.
24. Bartolomei S, Hoffman JR, Merni F, Stout JR. A comparison of traditional and block periodized strength training programs in trained athletes. *Journal of Strength and Conditioning Research*. 2014; 28(4):990-7.
25. Hall R. *A comparison of unilateral vs. bilateral leg strength training.* Eugene, Ore.;: Microform Publications, University of Oregon; 1985TY: GENGEN; Accession Number: SPH175914; Author: Hall, R. ; Language: English; General Notes: Thesis (M.S.) - University of Arizona, 1983; includes bibliography. Available from: Microform Publications, International Institute for Sport and Human Performance, University of Oregon, Eugene, OR.; Description: 1 microfiche (41 fr.) : neg., ill.; 11 x 15 cm.; Publication Type: Microforms; Thesis or dissertation; Update Code: 19981201.
26. Cormie P, McGuigan MR, Newton RU. Adaptations in athletic performance after ballistic power versus strength training. *Medicine & Science in Sports & Exercise*. 2010; 42(8):1582-98.
27. Baker DG, Newton RU. Adaptations in upper-body maximal strength and power output resulting from long-term resistance training in experienced strength-power athletes. *Journal of Strength and Conditioning Research*. 2006; 1;20(3):541-6.
28. Stanforth PR, Painter TL, Wilmore JH. Alterations in Concentric Strength Consequent to Powercise and Universal Gym Circuit Training. *Journal of Strength and Conditioning Research*. 1992; 6(4):249-55.
29. Rhea MR, Kenn JG, Dermody BM. Alterations in speed of squat movement and the use of accommodated resistance among college athletes training for power. *Journal of Strength and Conditioning Research.* 2009; 23(9):2645-2650.
30. Wilmore J. Alterations in strength, body composition and anthropometric measurements consequent to a 10-week weight training program. *Medicine and Science in Sports*. 1974; 6(2):133-8.
31. Rhea MR, Peterson MD, Oliverson JR, Ayllon FN, Potenziano BJ. An examination of training on the VertiMax resisted jumping device for improvements in lower body power in highly trained college athletes. *Journal of Strength and Conditioning Research.* 2008; 22(3):735-740.
32. Brown BS, Gorman DR, DiBrezzo R, Fort I. Anaerobic power changes following short term, task specific, dynamic and static overload training. *Journal of Strength and Conditioning Research*. 1988; 2(2):35-8.
33. Newton RU, McEvoy KP. Baseball throwing velocity: A comparison of medicine ball training and weight training. *Journal of Strength and Conditioning Research*. 1994; 8(3):198-203.
34. Bartolomei S, Stout JR, Fukuda DH, Hoffman JR, Merni F. Block vs. weekly undulating periodized resistance training programs in women. *Journal of Strength and Conditioning Research*. 2015; 29(10):2679-87.
35. Abt JP, Oliver JM, Nagai T, Sell TC, Lovalekar MT, Beals K, et al. Block-Periodized Training Improves Physiological and Tactically Relevant Performance in Naval Special Warfare Operators. *Journal of Strength and Conditioning Research.* 2016; 30(1):39-52.
36. Siegler J, Gaskill S, Ruby B. Changes evaluated in soccer-specific power endurance either with or without a 10-week, in-season, intermittent, high-intensity training protocol. *Journal of Strength and Conditioning Research.* 2003; 17(2):379-387.
37. Frost DM, Bronson S, Cronin JB, Newton RU. Changes in Maximal Strength, Velocity, and Power After 8 Weeks of Training With Pneumatic or Free Weight Resistance. *Journal of Strength and Conditioning Research.* 2016; 30(4):934-944.
38. Lloyd RS, Radnor JM, Croix MB, Cronin JB, Oliver JL. Changes in sprint and jump performances after traditional, plyometric, and combined resistance training in male youth pre-and post-peak height velocity. *Journal of Strength and Conditioning Research*. 2016; 30(5):1239-47.
39. de Hoyo M, Gonzalo-Skok O, Sañudo B, Carrascal C, Plaza-Armas JR, Camacho-Candil F, Otero-Esquina C. Comparative effects of in-season full-back squat, resisted sprint training, and plyometric training on explosive performance in U-19 elite soccer players. *Journal of Strength and Conditioning Research*. 2016; 30(2):368-77.
40. Mihalik JP, Libby JJ, Battaglini CL, McMurray RG. Comparing short-term complex and compound training programs on vertical jump height and power output. *Journal of Strength and Conditioning Research*. 2008; 22(1):47-53.
41. Teo SY, Newton MJ, Newton RU, Dempsey AR, Fairchild TJ. Comparing the effectiveness of a short-term vertical jump vs. weightlifting program on athletic power development. *Journal of Strength and Conditioning Research*. 2016; 30(10):2741-8.
42. Hoffman JR, Ratamess NA, Klatt M, Faigenbaum AD, Ross RE, Tranchina NM, McCurley RC, Kang J, Kraemer WJ. Comparison between different off-season resistance training programs in Division III American college football players. *Journal of Strength and Conditioning Research*. 2009; 23(1):11-19.
43. Bartolomei S, Hoffman JR, Stout JR, Zini M, Stefanelli C, Merni F. Comparison of Block Versus Weekly Undulating Periodization Models on Endocrine and Strength Changes in Male Athletes. *Kinesiology.* 2016; 48(1):71-78.
44. Schmidtbleicher D, Wirth K. Comparison of different strength trainings methods for the development of power. [Accession Number: SPHS-1059438; Conference: International Symposium on Biomechanics in Sports (24th : 2006 : Salzburg, Austria).
45. Rana SR, Chleboun GS, Gilders RM, Hagerman FC, Herman JR, Hikida RS, et al. Comparison of early phase adaptations for traditional strength and endurance, and low velocity resistance training programs in college-aged women. *Journal of Strength and Conditioning Research.* 2008; 22(1):119-127.
46. Harries SK, Lubans DR, Callister R. Comparison of resistance training progression models on maximal strength in sub-elite adolescent rugby union players. *Journal of Science and Medicine in Sport*. 2016; 19(2):163-9.
47. Bauer T, Thayer RE, Baras G. Comparison of training modalities for power development in the lower extremity. *Journal of Strength and Conditioning Research*. 1990; 4(4):115-21.
48. Aarskog R, Wisnes A, Wilhelmsen K, Skogen A, Bjordal JM. Comparison of Two Resistance Training Protocols, 6RM versus 12RM, to Increase the 1RM in Healthy Young Adults. A Single-Blind, Randomized Controlled Trial. *Physiotherapy Research International.* 2012; 17(3):179-186.
49. Hori N, Newton RU, Kawamori N, McGuigan MR, Andrews WA, Chapman DW, et al. Comparison of weighted jump squat training with and without eccentric braking. *Journal of Strength and Conditioning Research.* 2008; 22(1):54-65.
50. Robineau J, Lacome M, Piscione J, Bigard X, Babault N. Concurrent Training in Rugby Sevens: Effects of High-Intensity Interval Exercises. *International journal of sports physiology and performance.* 2017; 12(3):336-344.
51. Hansen KT, Cronin JB, Pickering SL, Newton MJ. Does cluster loading enhance lower body power development in preseason preparation of elite rugby union players?. *Journal of Strength and Conditioning Research*. 2011; 25(8):2118-26.
52. Milanovic Z, Sporis G, Trajkovic N, Sekulic D, James N, Vuckovic G. Does SAQ training improve the speed and flexibility of young soccer players? A randomized controlled trial. *Human Movement Science.* 2014; 38:197-208.
53. Radaelli R, Fleck SJ, Leite T, Leite RD, Pinto RS, Fernandes L, Simão R. Dose-response of 1, 3, and 5 sets of resistance exercise on strength, local muscular endurance, and hypertrophy. *Journal of Strength and Conditioning Research*. 2015; 29(5):1349-58.
54. Ataee J, Koozehchian MS, Kreider RB, Zuo L. Effectiveness of accommodation and constant resistance training on maximal strength and power in trained athletes. *PeerJ.* 2014; 2014(1) (pagination):Arte Number: e441. ate of Pubaton: 2014.
55. Kerksick CM, Wilborn CD, Campbell BI, Roberts MD, Rasmussen CJ, Greenwood M, Kreider RB. Early-phase adaptations to a split-body, linear periodization resistance training program in college-aged and middle-aged men. *Journal of Strength and Conditioning Research*. 2009; 23(3):962-71.
56. Wirth K, Keiner M, Hartmann H, Sander A, Mickel C. Effect of 8 weeks of free-weight and machine-based strength training on strength and power performance. *Journal of human kinetics*. 2016; 53:201.
57. Laurent C, Penzer F, Letroye B, Carpentier A, Baudry S, Duchateau J. Effect of a strength training method characterized by an incremental number of repetitions across sets and a very short rest interval. *Science and Sports.* 2016; 31(5):e115-e121.
58. Manolopoulos K, Gissis I, Galazoulas C, Manolopoulos E, Patikas D, Gollhofer A., et al. Effect of Combined Sensorimotor-Resistance Training on Strength, Balance, and Jumping Performance of Soccer Players. *Journal of Strength and Conditioning Research.* 2016; 30(1):53-59.
59. Koundourakis NE, Androulakis N, Spyridaki EC, Castanas E, Malliaraki N, Tsatsanis C, et al. Effect of different seasonal strength training protocols on circulating androgen levels and performance parameters in professional soccer players. *Hormones.* 2014; 13(1):104-118.
60. Coutts AJ, Murphy AJ, Dascombe BJ. Effect of direct supervision of a strength coach on measures of muscular strength and power in young rugby league players. *Journal of Strength and Conditioning Research.* 2004; 18(2):316-323.
61. Randell AD, Cronin JB, Keogh JWL, Gill ND, Pedersen MC. Effect of instantaneous performance feedback during 6 weeks of velocity-based resistance training on sport-specific performance tests. *Journal of Strength and Conditioning Research.* 2011; 25(1):87-93.
62. Bartolomei S, Hoffman JR, Stout JR, Merni F. Effect of Lower-Body Resistance Training on Upper-Body Strength Adaptation in Trained Men. *Journal of Strength and Conditioning Research.* 2018; 32(1):13-18.
63. Pareja-Blanco F, Rodriguez-Rosell D, Sanchez-Medina L, Gorostiaga EM, Gonzalez-Badillo JJ. Effect of movement velocity during resistance training on neuromuscular performance. *International Journal of Sports Medicine.* 2014; 35(11):916-924.
64. Channell BT, Barfield JP. Effect of Olympic and traditional resistance training on vertical jump improvement in high school boys. *Journal of Strength and Conditioning Research.* 2008; 22(5):1522-1527.
65. Korak JA, Paquette MR, Brooks J, Fuller DK, Coons JM. Effect of rest-pause vs. traditional bench press training on muscle strength, electromyography, and lifting volume in randomized trial protocols. *European journal of applied physiology*. 2017; 117(9):1891-6.
66. Blazevich AJ, Jenkins DG. Effect of the movement speed of resistance training exercise on sprint and strength performance in concurrently training elite junior sprinters. *Journal of Orthopaedic & Sports Physical Therapy.* 2002; 20(12):981-90.
67. Lamberth J, Hale B, Knight A, Boyd J, Luczak T. Effectiveness of a Six-Week Strength and Functional Training Program on Golf Performance. *International Journal of Golf Science.* 2013; 2(1):33-42.
68. Jimenez-Reyes P, Samozino P, Brughelli M, Morin JB. Effectiveness of an individualized training based on force-velocity profiling during jumping. *Frontiers in Physiology.* 2017; 7(JAN) (pagination):Arte Number: 677. ate of Pubaton: 2017.
69. Brown AC, Wells TJ, Schade ML, Smith DL, Fehling PC. Effects of Plyometric Training Versus Traditional Weight Training on Strength, Power, and Aesthetic Jumping Ability in Female Collegiate Dancers. *Journal of Dance Medicine & Science.* 2007; 11(2):38-44.
70. Paz-Franco A, Rey E, Barcala-Furelos R. Effects of 3 Different Resistance Training Frequencies on Jump, Sprint, and Repeated Sprint Ability Performances in Professional Futsal Players. *Journal of Strength and Conditioning Research.* 2017; 31(12):3343-3350.
71. Kim E, Dear A, Ferguson SL, Seo D, Bemben MG. Effects of 4 weeks of traditional resistance training vs. superslow strength training on early phase adaptations in strength, flexibility, and aerobic capacity in college-aged women. *Journal of Strength and Conditioning Research*. 2011; 25(11):3006-13.
72. Rodríguez-Rosell D, Franco-Márquez F, Pareja-Blanco F, Mora-Custodio R, Yáñez-García JM, González-Suárez JM, González-Badillo JJ. Effects of 6 weeks resistance training combined with plyometric and speed exercises on physical performance of pre-peak-height-velocity soccer players. *International journal of sports physiology and performance*. 2016; 11(2):240-6.
73. Hermassi S, Chelly MS, Tabka Z, Shephard RJ, Chamari K. Effects of 8-week in-season upper and lower limb heavy resistance training on the peak power, throwing velocity, and sprint performance of elite male handball players. *Journal of Strength and Conditioning Research.* 2011; 25(9):2424-2433.
74. Wong PL, Chamari K, Wisløff U. Effects of 12-week on-field combined strength and power training on physical performance among U-14 young soccer players. *Journal of Strength and Conditioning Research*. 2010; 24(3):644-52.
75. Harries SK, Lubans DR, Buxton A, MacDougall THJ, Callister R. Effects of 12-weeks resistance training on sprint and jump performance in competitive adolescent rugby union players. *Journal of Strength and Conditioning Research.* 2018; 32(10):2762-9.
76. Veliz RR, Requena B, Suarez-Arrones L, Newton RU, De Villarreal ES. Effects of 18-week in-season heavy-resistance and power training on throwing velocity, strength, jumping, and maximal sprint swim performance of elite male water polo players. *Journal of Strength and Conditioning Research*. 2014; 28(4):1007-14.
77. Manolopoulos E, Katis A, Manolopoulos K, Kalapotharakos V, Kellis E. Effects of a 10-week resistance exercise program on soccer kick biomechanics and muscle strength. *Journal of Strength and Conditioning Research.* 2013; 27(12):3391-3401.
78. Redondo JC, Alonso CJ, Sedano S, de Benito AM. Effects of a 12-week strength training program on experimented fencers' movement time. *Journal of Strength and Conditioning Research*. 2014; 28(12):3375-84.
79. Chelly MS, Fathloun M, Cherif N, Amar MB, Tabka Z, Van Praagh E. Effects of a back squat training program on leg power, jump, and sprint performances in junior soccer players. *Journal of Strength and Conditioning Research*. 2009; 23(8):2241-9.
80. Faigenbaum AD, McFarland JE, Keiper FB, Tevlin W, Ratamess NA, Kang J, Hoffman JR. Effects of a short-term plyometric and resistance training program on fitness performance in boys age 12 to 15 years. *Journal of sports science & medicine*. 2007; 6(4):519-525.
81. Contreras B, Vigotsky AD, Schoenfeld BJ, Beardsley C, McMaster DT, Reyneke JH, Cronin JB. Effects of a six-week hip thrust vs. front squat resistance training program on performance in adolescent males: a randomized controlled trial. *Journal of Strength and Conditioning Research*. 2017; 31(4):999-1008.
82. Inovero JG, Pagaduan JC. Effects of a Six-Week Strength Training and Upper Body Plyometrics in Male College Basketball Physical Education Students. *Sport Scientific & Practical Aspects.* 2015; 12(1):11-16.
83. Bourgeois FA, Gamble P, Gill ND, McGuigan MR. Effects of a six-week strength training programme on change of direction performance in youth team sport athletes. *Sports*. 2017; 5(4):83.
84. Vaara JP, Kokko J, Isoranta M, Kyrolainen H. Effects of Added Resistance Training on Physical Fitness, Body Composition, and Serum Hormone Concentrations During Eight Weeks of Special Military Training Period. *Journal of Strength and Conditioning Research.* 2015; 29:S168-S172.
85. Robbins DW, Young WB, Behm DG, Payne WR. Effects of agonist-antagonist complex resistance training on upper body strength and power development. *Journal of sports sciences.* 2009; 27(14):1617-1625.
86. Alvarez M, Sedano S, Cuadrado G, Redondo JC. Effects of an 18-week strength training program on low-handicap golfers' performance. *Journal of Strength and Conditioning Research.* 2012; 26(4):1110-1121.
87. Smart DJ, Gill ND. Effects of an off-season conditioning program on the physical characteristics of adolescent rugby union players. *Journal of Strength and Conditioning Research*. 2013; 27(3):708-17.
88. Newton RU, Kraemer WJ, Hakkinen K. Effects of ballistic training on preseason preparation of elite volleyball players. *Medicine and Science in Sports and Exercise.* 1999; 31(2):323-330.
89. Franco Márquez F, Rodríguez Rosell D, González Suárez JM, Pareja Blanco F, Mora Custodio R, Yáñez García JM, González Badillo JJ. Effects of combined resistance training and plyometrics on physical performance in young soccer players. *International Journal of Sports Medicine.* 2015.
90. Santos EJAM, Janeira MAAS. Effects of complex training on explosive strength in adolescent male basketball players. *Journal of Strength and Conditioning Research*. 2008; 22(3):903-9.
91. Hartmann H, Bob A, Wirth K, Schmidtbleicher D. Effects of different periodization models on rate of force development and power ability of the upper extremity. *Journal of Strength and Conditioning Research*. 2009; 23(7):1921-32.
92. Naclerio F, Faigenbaum AD, Larumbe-Zabala E, Perez-Bibao T, Kang J, Ratamess NA, et al. Effects of different resistance training volumes on strength and power in team sport athletes. *Journal of Strength and Conditioning Research.* 2013; 27(7):1832-1840.
93. Robinson JM, Stone MH, Johnson RL, Penland CM, Warren BJ, Lewis RD. Effects of different weight training exercise/rest intervals on strength, power, and high intensity exercise endurance. *Journal of Strength and Conditioning Research*. 1995; 9(4):216-21.
94. Wirth K, Keiner M, Szilvas E, Hartmann H, Sander A. Effects of eccentric strength training on different maximal strength and speed-strength parameters of the lower extremity. *Journal of Strength and Conditioning Research*. 2015; 29(7):1837-45.
95. Tomljanović M, Spasić M, Gabrilo G, Uljević O, Foretić N. Effects of Five Weeks of Functional Vs. Traditional Resistance Training on Anthropometric and Motor Performance Variables. / Efekti 5-Tjednog Funkcionalnoga i Tradicionalnogatreninga S OptereĆenjem Na Antropometrijskekarakteristike i MotoriČke Sposobnosti. *Kinesiology.* 2011; 43(2):145-154.
96. Gorostiaga EM, Izquierdo M, Iturralde P, Ruesta M, Ibanez J. Effects of heavy resistance training on maximal and explosive force production, endurance and serum hormones in adolescent handball players. *European journal of applied physiology and occupational physiology.* 1999; 80(5):485-493.
97. Janusevicius D, Snieckus A, Skurvydas A, Silinskas V, Trinkunas E, Cadefau JA, Kamandulis S. Effects of high velocity elastic band versus heavy resistance training on hamstring strength, activation, and sprint running performance. *Journal of sports science & medicine*. 2017; 16(2):239.
98. Pierce K, Rozenek R, Stone MH. Effects of high volume weight training on lactate, heart rate, and perceived exertion. *Journal of Strength and Conditioning Research*. 1993; 7(4):211-5.
99. Negra Y, Chaabene H, Hammami M, Hachana Y, Granacher U. Effects of high-velocity resistance training on athletic performance in prepuberal male soccer athletes. *Journal of Strength and Conditioning Researc*h. 2016; 30(12):3290-7.
100. Kubo K, Yata H, Kanehisa H, Fukunaga T. Effects of isometric squat training on the tendon stiffness and jump performance. *European journal of applied physiology.* 2006; 96(3):305-314.
101. Rodríguez-Rosell D, Torres-Torrelo J, Franco-Márquez F, González-Suárez JM, González-Badillo JJ. Effects of light-load maximal lifting velocity weight training vs. combined weight training and plyometrics on sprint, vertical jump and strength performance in adult soccer players. *Journal of science and medicine in sport*. 2017; 20(7):695-9.
102. Wirtz N, Zinner C, Doermann U, Kleinoeder H, Mester J. Effects of Loaded Squat Exercise with and without Application of Superimposed EMS on Physical Performance. *Journal of Sports Science and Medicine.* 2016; 15(1):26-33.
103. Hammami M, Negra Y, Billaut F, Hermassi S, Shephard RJ, Chelly MS. Effects of Lower-Limb Strength Training on Agility, Repeated Sprinting With Changes of Direction, Leg Peak Power, and Neuromuscular Adaptations of Soccer Players. *Journal of Strength and Conditioning Research.* 2018; 32(1):37-47.
104. Moss BM, Refsnes PE, Abildgaard A, Nicolaysen K, Jensen J. Effects of maximal effort strength training with different loads on dynamic strength, cross-sectional area, load-power and load-velocity relationships. *European journal of applied physiology and occupational physiology*. 1997; 75(3):193-9.
105. Karsten B, Larumb-Zabala E, Kandemir G, Hazir T, Klose A, Naclerio F. The effects of a 6-week strength training on critical velocity, anaerobic running distance, 30-M sprint and Yo-Yo intermittent running test performances in male soccer players. *PLoS ONE.* 2016; 11(3) (pagination):Arte Number: e0151448. ate of Pubaton: Marh 2016.
106. Bruhn S, Kullmann N, Gollhofer A. The Effects of a Sensorimotor Training and a Strength Training on Postural Stabilisation, Maximum Isometric Contraction and Jump Performance. *International Journal of Sports Medicine.* 2004; 25(1):56-60.
107. Zisis P. The effects of an 8 weeks plyometric training program or an explosive strength training program on the Jump-and-Reach Height of male amateur soccer players. *Journal of Physical Education and Sport.* 2013; 13(4):594-600.
108. Fry AC, Kraemer WJ, Weseman CA, Conroy BP, Gordon SE, Hoffman JR, Maresh CM. The effects of an off-season strength and conditioning program on starters and non-starters in women's intercollegiate volleyball. *Journal of Strength and Conditioning Research*. 1991; 5(4):174-81.
109. Mangine GT, Ratamess NA, Hoffman JR, Faigenbaum AD, Kang J., Chilakos A. The effects of combined ballistic and heavy resistance training on maximal lower- and upper-body strength in recreationally trained men. *Journal of Strength and Conditioning Research.* 2008; 22(1):132-139.
110. Apanukul S, Suwannathada S, Intiraporn C. The Effects of Combined Weight and Pneumatic Training to Enhance Power Endurance in Tennis Players. *Journal of Exercise Physiology Online.* 2015; 18(2):8-16.
111. Anderson CE, Sforzo GA, Sigg JA. The effects of combining elastic and free weight resistance on strength and power in athletes. *Journal of strength and conditioning research.* 2008; 22(2):567-574.
112. Pritchard HJ, Fink PW, Stannard SR. The Effects of Concentric/eccentric Training Versus Concentric Only Training on Peak Power and Functional Muscle Performance. *Journal of Australian Strength and Conditioning.* 2015; 23(6):71-75.
113. Lockie RG, Murphy AJ, Schultz AB, Knight TJ, de Jonge XA. The effects of different speed training protocols on sprint acceleration kinematics and muscle strength and power in field sport athletes. *Journal of Strength and Conditioning Research*. 2012; 26(6):1539-50.
114. Dolezal SM, Frese DL, Llewellyn TL. The Effects of Eccentric, Velocity-Based Training on Strength and Power in Collegiate Athletes. *International Journal of Exercise Science.* 2016; 9(5):657-666.
115. Hoff J, Almasbakk B. The effects of maximum strength training on throwing velocity and muscle strength in female team-handball players. *Journal of Strength and Conditioning Research.* 1995; 9(4):255-258.
116. Liu C, Chen CS, Ho WH, Fule RJ, Chung PH, Shiang TY. The effects of passive leg press training on jumping performance, speed, and muscle power. *Journal of Strength and Conditioning Research.* 2013; 27(6):1479-1486.
117. Herrick AB, Stone WJ. The effects of periodization versus progressive resistance exercise on upper and lower body strength in women. *Journal of Strength and Conditioning Research*. 1996 May 1;10(2):72-6.
118. Santos EJAM, Janeira MAAS. The effects of resistance training on explosive strength indicators in adolescent basketball players. *Journal of Strength and Conditioning Research*. 2012; 26(10):2641-7.
119. Smith RA, Martin GJ, Szivak TK, Comstock BA, Dunn-Lewis C, Hooper DR, Flanagan SD, Looney DP, Volek JS, Maresh CM, Kraemer WJ. The effects of resistance training prioritization in NCAA Division I Football summer training. *Journal of Strength and Conditioning Research*. 2014; 28(1):14-22.
120. Hoffman JR, Kraemer WJ, Fry AC, Deschenes M, Kemp M. The effects of self-selection for frequency of training in a winter conditioning program for football. *Journal of Strength and Conditioning Research*. 1990; 4(3):76-82.
121. Stalder MA, Noble BJ, Wilkinson JG. The effects of supplemental weight training for ballet dancers.  *Journal of Strength and Conditioning Research*. 1990; 4(3):95-102.
122. Cressey EM, West CA, Tiberio DP, Kraemer WJ, Maresh CM. The effects of ten weeks of lower-body unstable surface training on markers of athletic performance. *Journal of Strength and Conditioning Research.* 2007; 21(2):561-567.
123. Wirth K, Keiner M, Szilvas E, Hartmann H, Sander A. Effects of eccentric strength training on different maximal strength and speed-strength parameters of the lower extremity. *Journal of Strength and Conditioning Research*. 2015; 29(7):1837-45.
124. Sleivert GG, Backus RD, Wenger HA. The influence of a strength-sprint training sequence on multi-joint power output. *Medicine and science in sports and exercise.* 1995; 27(12):1655-1665.
125. Mazzetti SA, Kraemer WJ, Volek JS, Duncan ND, Ratamess NA, Gomez AL, et al. The influence of direct supervision of resistance training on strength performance. *Medicine and science in sports and exercise.* 2000; 32(6):1175-1184.
126. Bishop D, Jenkins DG. The influence of resistance training on the critical power function and time to fatigue at critical power. *Australian Journal of Science and Medicine in Sport.* 1996; 28(4):101-105.
127. Bloomfield J, Blanksby BA, Ackland TR, Allison GT. The influence of strength training on overhead throwing velocity of elite water polo players. *Australian Journal of Science and Medicine in Sport.* 1990; 22(3):63-67.
128. Petersen SR, Bagnall KM, Wenger HA, Reid DC, Castor WR, Quinney HA. The influence of velocity-specific resistance training on the in vivo torque-velocity relationship and the cross-sectional area of quadriceps femoris. *Journal of Orthopaedic & Sports Physical Therapy.* 1989; 10(11):456-462.
129. Hennessy LC, Watson AW. The interference effects of training for strength and endurance simultaneously. *Journal of Strength and Conditioning Research*. 1994; 8(1):12-9.
130. Wilson GJ, Newton RU, Murphy AJ, Humphries BJ. The optimal training load for the development of dynamic athletic performance. *Medicine and science in sports and exercise.* 1993; 25(11):1279-1286.
131. Schiotz MK, Potteiger JA, Huntsinger PG, Denmark LC. The short-term effects of periodized and constant-intensity training on body composition, strength, and performance. *Journal of Strength and Conditioning Research*. 1998; 12(3):173-8.
132. Secomb JL, Farley OR, Nimphius S, Lundgren L, Tran TT, Sheppard JM. The training-specific adaptations resulting from resistance training, gymnastics and plyometric training, and non-training in adolescent athletes. *International Journal of Sports Science & Coaching.* 2017; 12(6):762-773.
133. Sparkes R, Behm DG. Training adaptations associated with an 8-week instability resistance training program with recreationally active individuals. *Journal of Strength and Conditioning Research.* 2010; 24(7):1931-1941.
134. Blazevich AJ, Gill ND, Bronks R., Newton RU. Training-Specific Muscle Architecture Adaptation after 5-wk Training in Athletes. *Medicine and science in sports and exercise.* 2003; 35(12):2013-2022.
135. Speirs DE, Bennett MA, Finn CV, Turner AP. Unilateral vs. bilateral squat training for strength, sprints, and agility in academy rugby players. *Journal of Strength and Conditioning Research*. 2016; 30(2):386-92.
136. Rivière M, Louit L, Strokosch A, Seitz LB. Variable resistance training promotes greater strength and power adaptations than traditional resistance training in elite youth rugby league players. *Journal of Strength and Conditioning Research*. 2017; 31(4):947-55.
137. Palmieri GA. Weight training and repetition speed. *Journal of Strength and Conditioning Research*. 1987; 1(2):36-8.
138. Augustsson J, Esko A, Thomee R, Svantesson U. Weight training of the thigh muscles using closed vs. Open kinetic chain exercises: A comparison of performance enhancement. *Journal of Orthopaedic and Sports Physical Therapy.* 1998; 27(1):3-8.
139. Vanderka M, Novosád A. Weighted Squat Training with and without Counter Movement for Strength and Power Development. / Vplyv TrÉningu Drepov so ZÁŤaŽou, Bez a S Protipohybom Na Zmeny Úrovne SilovÝch a RÝchlostno-SilovÝch SchopnostÍ. *Acta Facultatis Educationis Physicae Universitatis Comenianae.* 2012; 52(1):21-27.
140. Hakkinen K, Newton RU, Gordon SE, McCormick M, Volek JS, Nindl BC, et al. Changes in muscle morphology, electromyographic activity, and force production characteristics during progressive strength training in young and older men. *Journals of Gerontology Series A-Biological Sciences & Medical Sciences.* 1998; 53(6):B415-23.
141. Andersen LL, Andersen JL, Zebis MK, Aagaard P. Early and late rate of force development: differential adaptive responses to resistance training?. *Scandinavian Journal of Medicine & Science in Sports.* 2010; 20(1):e162-169.
142. Iglesias-Soler E, Mayo X, Rio-Rodriguez D, Carballeira E, Farinas J, Fernandez-Delolmo M. Inter-repetition rest training and traditional set configuration produce similar strength gains without cortical adaptations. *Journal of sports sciences.* 2016; 34(15):1473-1484.
143. MacDonald CJ, Lamont HS, Garner JC. A comparison of the effects of 6 weeks of traditional resistance training, plyometric training, and complex training on measures of strength and anthropometrics. *Journal of Strength and Conditioning Research*. 2012; 26(2):422-31.
144. Kraemer WJ, Nindl BC, Ratamess NA, Gotshalk LA, Volek JS, Fleck SJ, et al. Changes in Muscle Hypertrophy in Women with Periodized Resistance Training. *Medicine and science in sports and exercise.* 2004; 36(4):697-708.
145. Andersen LL, Andersen JL, Magnusson SP, Suetta C, Madsen JL, Christensen LR, et al. Changes in the human muscle force-velocity relationship in response to resistance training and subsequent detraining. *Journal of applied physiology.* 2005; 99(1):87-94.
146. Bruhn S, Kullmann N, Gollhofer A. Combinatory effects of high-intensity-strength training and sensorimotor training on muscle strength. *International Journal of Sports Medicine.* 2006; 27(5):401-406.
147. Vossen JF, Kramer JF, Burke DG, Vossen DP. Comparison of dynamic push-up training and plyometric push-up training on upper-body power and strength. *Journal of Strength & Conditioning Research.* 2000; 14(3):248-253.
148. Moore E.W.G., Hickey M.S., Reiser II RF. Comparison of two twelve week off-season combined training programs on entry level collegiate soccer players' performance. *Journal of Strength and Conditioning Research.* 2005; 19(4):791-798.
149. McCarthy JP, Agre JC, Graf BK, Pozniak MA, Vailas AC. Compatibility of adaptive responses with combining strength and endurance training. *Medicine and science in sports and exercise.* 1995; 27(3):429-436.
150. Izquierdo-Gabarren M, Gonzalez De Txabarri Exposito R, Garcia-pallares J, Sanchez-medina L, De Villarreal ESS, Izquierdo M. Concurrent endurance and strength training not to failure optimizes performance gains. *Medicine & Science in Sports & Exercise.* 2010; 42(6):1191-1199.
151. Loturco I, Ugrinowitsch C, Tricoli V, Pivetti B, Roschel H. Different loading schemes in power training during the preseason promote similar performance improvements in Brazilian elite soccer players. *Journal of Strength and Conditioning Research.* 2013; 27(7):1791-1797.
152. Izquierdo M, Ibanez J, Gonzalez-Badillo JJ, Hakkinen K, Ratamess NA, Kraemer WJ, et al. Differential effects of strength training leading to failure versus not to failure on hormonal responses, strength, and muscle power gains. *Journal of applied physiology.* 2006; 100(5):1647-1656.
153. Jones MT. Effect of compensatory acceleration training in combination with accommodating resistance on upper body strength in collegiate athletes. *Open Access Journal of Sports Medicine.* 2014; 5:183-189.
154. Potteiger JA, Williford Jr HN, Blessing DL, Smidt J. Effect of two training methods on improving baseball performance variables. *Journal of Strength and Conditioning Research*. 1992; 6(1):2-6.
155. Izquierdo M, Hakkinen K, Ibanez J, Kraemer WJ, Gorostiaga EM. Effects of combined resistance and cardiovascular training on strength, power, muscle cross-sectional area, and endurance markers in middle-aged men. *European journal of applied physiology.* 2005; 94(1-2):70-75.
156. Kobal R, Loturco I, Barroso R, Gil S, Cuniyochi R, Ugrinowitsch C, et al. Effects Of Different Combinations Of Strength, Power, And Plyometric Training On The Physical Performance Of Elite Young Soccer Players. *Journal of Strength and Conditioning Research.* 2017; 31(6):1468-76.
157. Aagaard P, Simonsen EB, Trolle M, Bangsbo J, Klausen K. Effects of different strength training regimes on moment and power generation during dynamic knee extensions. *European journal of applied physiology and occupational physiology.* 1994; 69(5):382-386.
158. Kraemer WJ, Hakkinen K, Newton RU, Nindl BC, Volek JS, McCormick M, et al. Effects of heavy-resistance training on hormonal response patterns in younger vs. older men. *Journal of applied physiology.* 1999; 87(3):982-992.
159. Aminaei M, Yazdani S, Amirseifadini M. Effects of plyometric and cluster resistance training on explosive power and maximum strength in karate players. *International Journal of Applied Exercise Physiology*. 2017; 6(2):34-44.
160. Hunter JP, Marshall RN. Effects of power and flexibility training on vertical jump technique. *Medicine and science in sports and exercise.* 2002; 34(3):478-486.
161. Hong A, Hong S, Shin Y. Effects of resistance training on muscle strength, endurance, and motor unit according to ciliary neurotrophic factor polymorphism in male college students. *Journal of Sports Science & Medicine.* 2014; 13(3):680-688.
162. Lamas L, Ugrinowitsch C, Rodacki A, Pereira G., Mattos E.C.T., Kohn A.F., et al. Effects of strength and power training on neuromuscular adaptations and jumping movement pattern and performance. *Journal of Strength and Conditioning Research.* 2012; 26(12):3335-3344.
163. Izquierdo M, Hakkinen K, Ibanez J, Garrues M, Anton A, Zuniga A, et al. Effects of strength training on muscle power and serum hormones in middle-aged and older men. *Journal of applied physiology.* 2001; 90(4):1497-1507.
164. de Hoyo M, Sañudo B, Carrasco L, Domínguez-Cobo S, Mateo-Cortes J, Cadenas-Sánchez MM, Nimphius S. Effects of traditional versus horizontal inertial flywheel power training on common sport-related tasks. *Journal of human kinetics*. 2015; 47:155-167.
165. Pareja-Blanco F, Rodriguez-Rosell D, Sanchez-Medina L, Sanchis-Moysi J, Dorado C, Mora-Custodio R, et al. Effects of velocity loss during resistance training on athletic performance, strength gains and muscle adaptations. *Scandinavian Journal of Medicine and Science in Sports.* 2017; 27(7):724-735.
166. Arazi H, Asadi A, Roohi S. Enhancing muscular performance in women: Compound versus complex, traditional resistance and plyometric training alone. *Journal of Musculoskeletal Research.* 2014; 17(2) (pagination):Arte Number: 1450007. ate of Pubaton: 22 Jun 2014.
167. De Villarreal ES, Requena B, Izquierdo M, Gonzalez-Badillo JJ. Enhancing sprint and strength performance: combined versus maximal power, traditional heavy-resistance and plyometric training. *Journal of science and medicine in sport*. 2013; 16(2):146-50.
168. Laird RH, Elmer DJ, Barberio MD, Salom LP, Lee KA, Pascoe DD. Evaluation of Performance Improvements After Either Resistance Training or Sprint Interval-Based Concurrent Training. *Journal of Strength and Conditioning Research.* 2016; 30(11):3057-3065.
169. Fink J, Kikuchi N, Yoshida S, Terada K, Nakazato K. Impact of high versus low fixed loads and non-linear training loads on muscle hypertrophy, strength and force development. *Springerplus.* 2016; 5(1):698.
170. Ignjatovic A., Radovanovic D., Stankovic R., Markovic Z., Kocic J. Influence of resistance training on cardiorespiratory endurance and muscle power and strength in young athletes. *Acta Physiologica Hungarica.* 2011; 98(3):305-312.
171. Kraemer WJ, Ratamess N, Fry AC, Triplett-Mcbride T, Koziris LP, Bauer JA, Lynch JM, Fleck SJ. Influence of resistance training volume and periodization on physiological and performance adaptations in collegiate women tennis players. *American Journal of Sports Medicine*. 2000; 28(5):626-33.
172. Talpey SW, Young WB, Saunders N. Is nine weeks of complex training effective for improving lower body strength, explosive muscle function, sprint and jumping performance? *International Journal of Sports Science and Coaching.* 2016; 11(5):736-745.
173. Berger R. Effect of varied weight training programs on strength. *Research Quarterly. American Association for Health, Physical Education and Recreation*. 1962; 33(2):168-81.
174. Dasteridis G, Pilianidis T, Mantzouranis N. The Effect of Different Strength Training Programs on Young Athletes' Sprint Performance. *Studies in Physical Culture & Tourism.* 2011; 18(2):141-147.
175. Whitley JD, Smith LE. Influence of three different training programs on strength and speed of a limb movement. *Research quarterly.* 1966; 37(1):132-142.
176. Jackson A, Jackson T, Hnatek J, West J. Strength development: Using functional isometrics in an isotonic strength training program. *Research quarterly for exercise and sport.* 1985; 56(3):234-237.
177. Kruszewski M, Kruszewski A, Kruszewski B. Changes in Relative and Absolute Force Measured during Powerlifting After a 4-Week Training using the Weightlifting Method and Stick Isometric (Mixed) Method. *Medicina Sportiva.* 2008; 12(2):41-45.
178. Clutch D, Wilton M, McGown C, Bryce GR. Effect of depth jumps and weight training on leg strength and vertical jump. *Research Quarterly for Exercise & Sport.* 1983; 54(1):5-10.
179. Bell GJ, Petersen SR, Quinney HA, Wenger HA. The effect of velocity-specific strength training on peak torque and anaerobic rowing power. *Journal of sports sciences.* 1989; 7(3):205-214.
180. Rutherford OM, Greig CA, Sargeant AJ, Jones DA. Strength training and power output: transference effects in the human quadriceps muscle. *Journal of sports sciences.* 1986; 4(2):101-107.
181. Doherty TJ, Campagna PD. The effects of periodized velocity-specific resistance training on maximal and sustained force production in women. *Journal of sports sciences.* 1993; 11(1):77-82.
182. Delecluse C, Van Coppenolle HE, Willems EU, Van Leemputte M, Diels R, Goris MA. Influence of high-resistance and high-velocity training on sprint performance. *Medicine and science in sports and exercise*. 1995; 27(8):1203-9.
183. O'Hagan FT, Sale DG, MacDougall JD, Garner SH. Comparative effectiveness of accommodating and weight resistance training modes. *Medicine and science in sports and exercise*. 1995; 27(8):1210-9.
184. Pesta D, Thaler A, Hoppel F, Macek C, Schocke M, Burtscher M. Effects of a 10-week conventional strength training program on lower leg muscle performance in adolescent boys compared to adults. *Journal of sports medicine and physical fitness*. 2014; 54(2):147-53.
185. Spineti J, Figueiredo T, Assis M, Miranda H, Simão R. Comparison between traditional strength training and complex contrast training on repeated sprint ability and muscle architecture in elite soccer players. *Journal of sports medicine and physical fitness*. 2015; 56(11):1269-78.
186. Voigt M, Klausen K. Changes in muscle strength and speed of an unloaded movement after various training programmes. *European journal of applied physiology and occupational physiology.* 1990; 60(5):370-376.
187. McMaster D, Gill N, McGuigan M, Cronin J. Effects of Complex Strength and Ballistic Training on Maximum Strength, Sprint Ability and Force-Velocity-Power Profiles of Semi-Professional Rugby Union Players. *Journal of Australian Strength and Conditioning.* 2014; 22(1):17-30.
188. Petersen TS. *Effects of slide board training on the lateral movement of college-aged football players.* Eugene, Or.;: Microform Publications, University of Oregon; 2000TY: GENGEN; Accession Number: SPHS-673351; Author: Petersen, T. S. ; Language: English; General Notes: Thesis (M.S.)University of Wisconsin, La Crosse, 2000; includes bibliographical references.; Description: 1 microfiche (97 fr.) : negative, ill. ; 11 x 15 cm.; Database Subset: ID; Publication Type: Microforms; Thesis or dissertation; Update Code: 20010401.
189. Ishoi L, Holmich P, Aagaard P, Thorborg K, Bandholm T, Serner A. Effects of the Nordic Hamstring exercise on sprint capacity in male football players: a randomized controlled trial. *Journal of sports sciences.* 2017; 1-10.
190. Housh T.J., Housh D.J., Weir J.P., Weir LL. Effects of unilateral concentric-only dynamic constant external resistance training. *International Journal of Sports Medicine.* 1996; 17(5):338-343.
191. Perez-Gomez J, Olmedillas H, Delgado-Guerra S, Royo IA, Vicente-Rodriguez G, Ortiz RA, et al. Effects of weight lifting training combined with plyometric exercises on physical fitness, body composition, and knee extension velocity during kicking in football. *Applied Physiology, Nutrition and Metabolism.* 2008; 33(3):501-510.
192. Brazell-Roberts JV, Thomas LE. Effects of weight training frequency on the self-concept of college females. *Journal of Strength and Conditioning Research*. 1989; 3(2):40-3.
193. Otto III WH, Coburn JW, Brown LE, Spiering BA. Effects of weightlifting vs. kettlebell training on vertical jump, strength, and body composition. *Journal of Strength and Conditioning Research*. 2012; 26(5):1199-202.
194. Tanimoto M, Sanada K, Yamamoto K, Kawano H, Gando Y, Tabata I, et al. Effects of whole-body low-intensity resistance training with slow movement and tonic force generation on muscular size and strength in young men. *Journal of Strength and Conditioning Research.* 2008; 22(6):1926-1938.
195. Moraes E, Fleck SJ, Dias MR, Simão R. Effects on strength, power, and flexibility in adolescents of nonperiodized vs. daily nonlinear periodized weight training. *Journal of Strength and Conditioning Research*. 2013; 27(12):3310-21.
196. Andersen V, Fimland MS, Kolnes MK, Saeterbakken AH. Elastic bands in combination with free weights in strength training: Neuromuscular effects. *Journal of Strength and Conditioning Research*. 2015; 29(10):2932-40.
197. Fyfe JJ, Bartlett JD, Hanson ED, Stepto NK, Bishop DJ. Endurance training intensity does not mediate interference to maximal lower-body strength gain during short-term concurrent training. *Frontiers in Physiology.* 2016; 7(NOV) (pagination):Arte Number: 487. ate of Pubaton: 03 No 2016.
198. De Villarreal ESS, Izquierdo M, Gonzalez-Badillo JJ. Enhancing jump performance after combined vs. maximal power, heavy-resistance, and plyometric training alone. *Journal of Strength and Conditioning Research.* 2011; 25(12):3274-3281.
199. De Villarreal ES, Suarez-Arrones L, Requena B, Haff GG, Veliz RR. Enhancing performance in professional water polo players: dryland training, in-water training, and combined training. *Journal of Strength and Conditioning Research*. 2015; 29(4):1089-1097.
200. Lyttle AD, Wilson GJ, Ostrowski KJ. Enhancing performance: maximal power versus combined weights and plyometrics training. *Journal of Strength and Conditioning Research.* 1996; 10(3):173-179.
201. Fatouros IG, Jamurtas AZ, Leontsini D, Taxildaris K, Aggelousis N, Kostopoulos N, Buckenmeyer P. Evaluation of plyometric exercise training, weight training, and their combination on vertical jumping performance and leg strength. *Journal of Strength and Conditioning Research*. 2000; 14(4):470-6.
202. Jenkins ND, Housh TJ, Buckner SL, Bergstrom HC, Smith CM, Cochrane KC, Hill EC, Miramonti AA, Schmidt RJ, Johnson GO, Cramer JT. Four weeks of high-versus low-load resistance training to failure on the rate of torque development, electromechanical delay, and contractile twitch properties. *Journal of musculoskeletal & neuronal interactions*. 2016; 16(2):135-144.
203. Maroto-Izquierdo S, García-López D, de Paz JA. Functional and muscle-size effects of flywheel resistance training with eccentric-overload in professional handball players. *Journal of human kinetics*. 2017; 60:133-143.
204. O’Shea K, O’Shea JP. Functional isometrics weight training: Its effects on dynamic and static strength. *Journal of Strength and Conditioning Research.* 1989; 3(2):30-33.
205. Giorgi A, Wilson GJ, Weatherby RP, Murphy AJ. Functional isometric weight training: its effects on the development of muscular function and the endocrine system over an 8-week training period. *Journal of Strength and Conditioning Research*. 1998; 12(1):18-25.
206. Fimland MS, Helgerud J, Gruber M, Leivseth G, Hoff J. Functional maximal strength training induces neural transfer to single-joint tasks. *European journal of applied physiology.* 2009; 107(1):21-29.
207. Oliver JM, Jagim AR, Sanchez AC, Mardock MA, Kelly KA, Meredith HJ, et al. Greater gains in strength and power with intraset rest intervals in hypertrophic training. *Journal of Strength and Conditioning Research.* 2013; 27(11):3116-3131.
208. Loturco I, Pereira LA, Kobal R, Zanetti V, Gil S, Kitamura K, Abad CC, Nakamura FY. Half-squat or jump squat training under optimum power load conditions to counteract power and speed decrements in Brazilian elite soccer players during the preseason. *Journal of sports sciences*. 2015; 33(12):1283-92.
209. Ayers JL, DeBeliso M, Sevene TG, Adams KJ. Hang cleans and hang snatches produce similar improvements in female collegiate athletes. *Biology of Sport.* 2016; 33(3):251-256.
210. Dæhlin TE, Haugen OC, Haugerud S, Hollan I, Raastad T, Rønnestad BR. Improvement of ice hockey players’ on-ice sprint with combined plyometric and strength training. *International journal of sports physiology and performance*. 2017; 12(7):893-900.
211. Ahmed TA. Improving musculoskeletal fitness and the performance enhancement of basketball skills through neuromuscular training program. Journal of Human Sport and Exercise. 2015;10(3):795-804.
212. Neils CM, Udermann BE, Brice GA, Winchester JB, McGuigan MR. Influence of Contraction Velocity in Untrained Individuals Over the Initial Early Phase of Resistance Training. *Journal of Strength and Conditioning Research.* 2005; 19(4):883-887.
213. Franchini E, Branco BM, Agostinho MF, Calmet M, Candau R. Influence of linear and undulating strength periodization on physical fitness, physiological, and performance responses to simulated judo matches*. Journal of Strength and Conditioning Research*. 2015; 29(2):358-67.
214. Hartmann H, Wirth K, Klusemann M, Dalic J, Matuschek C, Schmidtbleicher D. Influence of squatting depth on jumping performance. *Journal of Strength and Conditioning Research.* 2012; 26(12):3243-3261.
215. Otero-Esquina C, de HL, Gonzalo-Skok Ó, Domínguez-Cobo S, Sánchez H. Is strength-training frequency a key factor to develop performance adaptations in young elite soccer players? *European Journal of Sport Science.* 2017; 17(10):1241-1251.
216. Shalfawi SAI, Haugen T, Jakobsen TA, Enoksen E, Tønnessen E. The effect of combined resisted agility and repeated sprint training vs. strength training on female elite soccer players. *Journal of Strength and Conditioning Research*. 2013; 27(11):2966-72.
217. Tasi YJ, Liu GC, Chen CY, Huang C. The effect of different plyometric-squat training on taekwondo power development in the lower extremity. [Accession Number: SPHS-162162; Author: Tasi, Y.J. Author: Liu, G.C. Author: Chen, C.Y. Author: Huang, C. ; Corporate Author: International Society of Biomechanics in Sports; Conference: International Symposium on Biomechanics in Sports (17th : 1999 : Perth, Western Australia).; No. of Pages: 4; Language: English; Parent Item: SPHS-163221; References: 9; Database Subset: ID; Publication Type: Book Analytic; Update Code: 20000301; SIRC Article No.: S-162162]. Reproduced ; 1999.
218. Lopes CR, Aoki MS, Crisp AH, de Mattos RS, Lins MA, da Mota GR, Schoenfeld BJ, Marchetti PH. The effect of different resistance training load schemes on strength and body composition in trained men. *Journal of human kinetics*. 2017; 58:177-186.
219. Hawkins SB, Doyle TL, McGuigan MR. The effect of different training programs on eccentric energy utilization in college-aged males. *Journal of Strength and Conditioning Research*. 2009; 23(7):1996-2002.
220. Lee A, Craig BW, Lucas J, Pohlman R, Stelling H. The effect of endurance training, weight training and a combination of endurance and weight training upon the blood lipid profile of young male subjects. *Journal of Strength and Conditioning Research*. 1990; 4(3):68-75.
221. Alemdaroglu U, Dundar U, Koklu Y, Asci A, Findikoglu G. The effect of exercise order incorporating plyometric and resistance training on isokinetic leg strength and vertical jump performance: A comparative study. *Isokinetics and Exercise Science.* 2013; 21(3):211-217.
222. Hermassi S, Chelly MS, Fathloun M, Shephard RJ. The effect of heavy-vs. moderate-load training on the development of strength, power, and throwing ball velocity in male handball players. *Journal of Strength and Conditioning Research*. 2010; 24(9):2408-18.
223. Moir G, Sanders R, Button C, Glaister M. The effect of periodized resistance training on accelerative sprint performance. *Sports Biomechanics.* 2007; 6(3):285-300.
224. Arazi H, Khanmohammadi A, Asadi A, Haff GG. The effect of resistance training set configuration on strength, power, and hormonal adaptation in female volleyball players*. Applied physiology, nutrition, and metabolism*. 2018;43(2):154-64.
225. Wenzel RR, Perfetto EM. The effect of speed versus non-speed training in power development. *Journal of Applied Sport Science Research.* 1992; 6(2):82-87.
226. Hammami M, Negra Y, Shephard RJ, Chelly MS. The effect of standard strength vs. contrast strength training on the development of sprint, agility, repeated change of direction, and jump in junior male soccer players. *Journal of Strength and Conditioning Research*. 2017; 31(4):901-12.
227. Beattie K, Carson BP, Lyons M, Rossiter A, Kenny IC. The Effect of Strength Training on Performance Indicators in Distance Runners. *Journal of Strength and Conditioning Research.* 2017; 31(1):9-23.
228. Jakobsen MD, Sundstrup E, Randers MB, Kjær M, Andersen LL, Krustrup P, Aagaard P. The effect of strength training, recreational soccer and running exercise on stretch–shortening cycle muscle performance during countermovement jumping. *Human movement science*. 2012; 31(4):970-86.
229. Young WB, Bilby GE. The effect of voluntary effort to influence speed of contraction on strength, muscular power, and hypertrophy development. *Journal of Strength & Conditioning Research.* 1993; 7(3):172-178.
230. Ostrowski KJ, Wilson GJ, Weatherby R, Murphy PW, Lyttle AD. The effect of weight training volume on hormonal output and muscular size and function. *Journal of Strength and Conditioning Research*. 1997; 11:148-54.
231. Holcomb WR, Lander JE, Rutland RM, Wilson GD. The effectiveness of a modified plyometric program on power and the vertical jump. *Journal of Strength and Conditioning Research.* 1996; 10(2):89-92.
232. Rhea MR, Peterson MD, Lunt KT, Ayllon FN. The effectiveness of resisted jump training on the VertiMax in high school athletes. *Journal of Strength and Conditioning Research.* 2008; 22(3):731-734.
233. Shoepe TC, Ramirez DA, Rovetti RJ, Kohler DR, Almstedt HC. The Effects of 24 weeks of Resistance Training with Simultaneous Elastic and Free Weight Loading on Muscular Performance of Novice Lifters. *Journal of Human Kinetics.* 2011; 29:93-106.
234. Alcaraz PE, Elvira JLL, Palao JM. Kinematic, strength, and stiffness adaptations after a short-term sled towing training in athletes. *Scandinavian Journal of Medicine and Science in Sports.* 2014; 24(2):279-290.
235. Caruso JF, Coday MA, Ramsey CA, Griswold SH, Polanski DW, Drummond JL, et al. Leg and calf press training modes and their impact on jump performance adaptations. *Journal of strength and conditioning research / National Strength & Conditioning Association.* 2008; 22(3):766-772.
236. Torres-Torrelo J, Rodriguez-Rosell D, Gonzalez-Badillo JJ. Light-load maximal lifting velocity full squat training program improves important physical and skill characteristics in futsal players. *Journal of sports sciences.* 2017; 35(10):967-975.
237. Keiner M, Sander A, Wirth K, Schmidtbleicher D. Long-term strength training effects on change-of-direction sprint performance. *Journal of Strength and Conditioning Research*. 2014; 28(1):223-31.
238. Hong J, Smith JD, Ross CN, Lee S. Low volume progressive single set of resistance training is as effective as high volume multiple sets of resistance protocol on muscle strength and power. *International Journal of Applied Sports Sciences.* 2015; 27(1):33-42.
239. Kikuchi N, Nakazato K. Low-load bench press and push-up induce similar muscle hypertrophy and strength gain. *Journal of Exercise Science & Fitness.* 2017; 15(1):37-42.
240. Marx JO, Ratamess NA, Nindl BC, Gotshalk LA, Volek JS, Dohi K, et al. Low-volume circuit versus high-volume periodized resistance training in women. *Medicine and science in sports and exercise.* 2001; 33(4):635-643.
241. Tillin N, Folland J. Maximal and explosive strength training elicit distinct neuromuscular adaptations, specific to the training stimulus. *European journal of applied physiology.* 2014; 114(2):365-374.
242. Cherif M, Chtourou H, Souissi N, Aouidet A, Chamari K. Maximal power training induced different improvement in throwing velocity and muscle strength according to playing positions in elite male handball players. *Biology of Sport.* 2016; 33(4):393-398.
243. Coyne JO, Tran TT, Secomb JL, Lundgren LE, Farley OR, Newton RU, et al. Maximal Strength Training Improves Surfboard Sprint and Endurance Paddling Performance in Competitive and Recreational Surfers. *Journal of Strength and Conditioning Research.* 2017; 31(1):244-253.
244. Heggelund J, Fimland MS, Helgerud J, Hoff J. Maximal strength training improves work economy, rate of force development and maximal strength more than conventional strength training. *European journal of applied physiology*. 2013; 113(6):1565-73.
245. Smilios I, Sotiropoulos K, Christou M, Douda H, Spaias A, Tokmakidis SP. Maximum power training load determination and its effects on load-power relationship, maximum strength, and vertical jump performance. *Journal of Strength and Conditioning Research.* 2013; 27(5):1223-1233.
246. Newton RU, Hakkinen K, Hakkinen A, McCormick M, Volek J, Kraemer WJ. Mixed-methods resistance training increases power and strength of young and older men. *Medicine and science in sports and exercise.* 2002; 34(8):1367-1375.
247. Zourdos MC, Jo E, Khamoui AV, Lee SR, Park BS, Ormsbee MJ, et al. Modified Daily Undulating Periodization Model Produces Greater Performance Than a Traditional Configuration in Powerlifters. *Journal of Strength and Conditioning Research.* 2016; 30(3):784-791.
248. Vissing K, Brink M, Lønbro S, Sørensen H, Overgaard K, Danborg K, Mortensen J, Elstrøm OL, Rosenhøj N, Ringgaard S, Andersen JL. Muscle adaptations to plyometric vs. resistance training in untrained young men. *Journal of Strength and Conditioning Research*. 2008; 22(6):1799-810.
249. Toumi H, Best TM, Martin A, Poumarat G. Muscle plasticity after weight and combined (weight + jump) training. *Medicine and Science in Sports and Exercise.* 2004; 36(9):1580-1588.
250. Stasinaki AN, Gloumis G, Spengos K, Blazevich AJ, Zaras N., Georgiadis G., et al. Muscle Strength, Power, and Morphologic Adaptations After 6 Weeks of Compound vs. Complex Training in Healthy Men. *Journal of Strength and Conditioning Research.* 2015; 29(9):2559-2569.
251. Campos GER, Luecke TJ, Wendeln HK, Toma K, Hagerman FC, Murray TF, et al. Muscular adaptations in response to three different resistance-training regimens: Specificity of repetition maximum training zones. *European journal of applied physiology.* 2002; 88(1-2):50-60.
252. Vikne H, Refsnes PE, Ekmark M, Medbo JI, Gundersen V, Gundersen K. Muscular performance after concentric and eccentric exercise in trained men. *Medicine and science in sports and exercise.* 2006; 38(10):1770-1781.
253. Judge L, Moreau C, Burke J. Neural adaptations with sport-specific resistance training in highly skilled athletes. *Journal of Sports Sciences*. 2003; 21(5):419-27.
254. Häkkinen K, Alen M, Kraemer WJ, Gorostiaga E, Izquierdo M, Rusko H, Mikkola J, Häkkinen A, Valkeinen H, Kaarakainen E, Romu S. Neuromuscular adaptations during concurrent strength and endurance training versus strength training. *European journal of applied physiology*. 2003; 89(1):42-52.
255. Mikkola J, Rusko H, Izquierdo M, Gorostiaga EM, Hakkinen K. Neuromuscular and cardiovascular adaptations during concurrent strength and endurance training in untrained men. *International Journal of Sports Medicine.* 2012; 33(9):702-710.
256. Romero-Arenas S, Ruiz R, Vera-Ibanez A, Colomer-Poveda D, Guadalupe-Grau A, Marquez G. Neuromuscular and Cardiovascular Adaptations in Response to High-Intensity Interval Power Training. *Journal of Strength and Conditioning Research.* 2018; 32(1):130-138.
257. Ullrich B, Pelzer T, Oliveira S, Pfeiffer M. Neuromuscular responses to short-term resistance training with traditional and daily undulating periodization in adolescent elite judoka. *Journal of Strength and Conditioning Research*. 2016; 30(8):2083-99.
258. Myer GD, Ford KR, Palumbo JP, Hewett TE. Neuromuscular training improves performance and lower-extremity biomechanics in female athletes. *Journal of Strength and Conditioning Research.* 2005; 19(1):51-60.
259. Arabatzi F, Kellis E. Olympic weightlifting training causes different knee muscle-coactivation adaptations compared with traditional weight training. *Journal of Strength and Conditioning Research.* 2012; 26(8):2192-2201.
260. Jolley RI, Goodwin JE, Cleather DJ. Peak Power Output in the Bench Pull Is Maximized After Four Weeks of Specific Power Training. *Journal of Strength and Conditioning Research.* 2016; 30(4):966-972.
261. Baker D, Wilson G, Carlyon R. Periodization: the effect on strength of manipulating volume and intensity. *Journal of Strength and Conditioning Research (Allen Press Publishing Services Inc.).* 1994; 8(4):235-242.
262. Groves BR, Gayle RC. Physiological changes in male basketball players in year-round strength training. *Journal of Strength and Conditioning Research.* 1993; 7(1):30-33.
263. Kraemer WJ, Hakkinen K, Triplett-McBride NT, Fry AC, Koziris LP, Ratamess NA, et al. Physiological changes with periodized resistance training in women tennis players. *Medicine and science in sports and exercise.* 2003; 35(1):157-168.
264. Cormie P, McCaulley GO, McBride JM. Power versus strength-power jump squat training: influence on the load-power relationship. *Medicine and science in sports and exercise*. 2007; 39(6):996-1003.
265. Voelzke M, Stutzig N, Thorhauer HA, Granacher U. Promoting lower extremity strength in elite volleyball players: Effects of two combined training methods. *Journal of Science and Medicine in Sport.* 2012; 15(5):457-462.
266. Munn J, Herbert RD, Hancock MJ, Gandevia SC. Resistance training for strength: effect of number of sets and contraction speed. *Medicine and science in sports and exercise*. 2005; 37(9):1622-1626.
267. Mangine GT, Hoffman JR, Wang R, Gonzalez AM, Townsend JR, Wells AJ, Jajtner AR, Beyer KS, Boone CH, Miramonti AA, LaMonica MB. Resistance training intensity and volume affect changes in rate of force development in resistance-trained men. *European journal of applied physiology*. 2016; 116(11):2367-74.
268. Muehlbauer T, Gollhofer A, Granacher U. Sex-related effects in strength training during adolescence: A pilot study. *Perceptual and motor skills.* 2012; 115(3):953-968.
269. Winwood PW, Buckley JJ. Short Term Effects of Resistance Training Modalities on Performance Measures in Male Adolescents. *Journal of Strength and Conditioning Research.* 2019; 33(3):641-50.
270. Herrero AJ, Martin J, Martin T, Abadia O, Fernandez B, GarciaLopez D. Short-term effect of strength training with and without superimposed electrical stimulation on muscle strength and anaerobic performance. A randomized controlled trial. Part I. *Journal of Strength and Conditioning Research.* 2010; 24(6):1609-1615.
271. Alves JVM, Rebelo AN, Abrantes C, Sampaio J. Short-term effects of complex and contrast training in soccer players' vertical jump, sprint, and agility abilities. *Journal of Strength and Conditioning Research.* 2010; 24(4):936-941.
272. Cavaco B, Sousa N, Dos Reis VM, Garrido N, Saavedra F, Mendes R, Vilaça-Alves J. Short-term effects of complex training on agility with the ball, speed, efficiency of crossing and shooting in youth soccer players. *Journal of human kinetics*. 2014; 43:105.
273. Ronnestad BR, Kvamme NH, Sunde A, Raastad T. Short-term effects of strength and plyometric training on sprint and jump performance in professional soccer players. *Journal of Strength and Conditioning Research*. 2008; 22(3):773-80.
274. Tricoli V, Lamas L, Carnevale R, Ugrinowitsch C. Short-term effects on lower-body functional power development: weightlifting vs. vertical jump training programs. *Journal of Strength and Conditioning Research*. 2005; 19(2):433-7.
275. Sanborn K, Boros R, Hruby J, Schilling B, O'Bryant HS, Johnson RL, Hoke T, Stone ME, Stone MH. Short-term performance effects of weight training with multiple sets not to failure vs. a single set to failure in women. *Journal of Strength and Conditioning Research*. 2000; 14(3):328-31.
276. Kijowksi KN, Capps CR, Goodman CL, Erickson TM, Knorr DP, Triplett NT, Awelewa OO, McBride JM. Short-term resistance and plyometric training improves eccentric phase kinetics in jumping. *Journal of Strength and Conditioning Research*. 2015; 29(8):2186-96.
277. Farup J, Sørensen H, Kjølhede T. Similar changes in muscle fiber phenotype with differentiated consequences for rate of force development: endurance versus resistance training. *Human movement science*. 2014; 34:109-19.
278. Alcaraz PE, Perez-Gomez J, Chavarrias M, Blazevich AJ. Similarity in adaptations to high-resistance circuit vs. traditional strength training in resistance-trained men. *Journal of Strength and Conditioning Research.* 2011; 25(9):2519-2527.
279. Palmer T, Uhl TL, Howell D, Hewett TE, Viele K, Mattacola CG. Sport-specific training targeting the proximal segments and throwing velocity in collegiate throwing athletes. J*ournal of Athletic Training*. 2015; 50(6):567-77.
280. Knox A, Sculthorpe N, Baker JS, Grace F. Strength adaptation to squat exercise is different between Caucasian and South Asian novice exercisers. *Research in Sports Medicine*. 2017; 25(3):373-83.
281. Marshall P.W.M., McEwen M., Robbins DW. Strength and neuromuscular adaptation following one, four, and eight sets of high intensity resistance exercise in trained males. *European journal of applied physiology.* 2011; 111(12):3007-3016.
282. Painter KB, Haff GG, Ramsey MW, McBride J, Triplett T., Sands WA, et al. Strength gains: Block versus daily undulating periodization weight training among track and field athletes. *International Journal of Sports Physiology and Performance.* 2012; 7(2):161-169.
283. Delecluse C, Roelants M, Verschueren S. Strength increase after whole-body vibration compared with resistance training. *Medicine & Science in Sports & Exercise.* 2003; 35(6):1033-1041.
284. Manolopoulos E, Papadopoulos C, Salonikidis K, Katartzi E, Poluha S. Strength training effects on physical conditioning and instep kick kinematics in young amateur soccer players during preseason. *Perceptual and motor skills.* 2004; 99(2):701-710.
285. Folland JP, Hawker K, Leach B, Little T, Jones DA. Strength training: Isometric training at a range of joint angles versus dynamic training. *Journal of sports sciences.* 2005; 23(8):817-824.
286. Stone WJ, Coulter SP. Strength/endurance effects from three resistance training protocols with women. *Journal of Strength and Conditioning Research*. 1994; 8(4):231-4.
287. Blakey JB, Southard D. The combined effects of weight training and plyometrics on dynamic leg strength and leg power. *Journal of Applied Sport Science Research.* 1987; 1(1):14-16.
288. Parnow A, Derakhshandeh S, Hosseini A. The Effect of 4-week Difference Training Methods on Some Fitness Variables in Youth Handball Players. *International Journal of Applied Exercise Physiology*. 2016; 5(3).
289. Mills JD, Taunton JE, Mills WA. The effect of a 10-week training regimen on lumbo-pelvic stability and athletic performance in female athletes: A randomized-controlled trial. *Physical Therapy in Sport.* 2005; 6(2):60-66.
290. Kotzamanidis C, Chatzopoulos DI, Michailidis C, Papaiakovou G, Patikas DI. The effect of a combined high-intensity strength and speed training program on the running and jumping ability of soccer players. *Journal of Strength and Conditioning Research*. 2005; 19(2):369-75.
291. Kubo K, Morimoto M, Komuro T, Yata H, Tsunoda N, Kanehisa H, Fukunaga T. Effects of plyometric and weight training on muscle-tendon complex and jump performance. *Medicine and science in sports and exercise*. 2007; 39(10):1801-10.
292. Damasceno MV, Lima-Silva AE, Pasqua LA, Tricoli V, Duarte M, Bishop DJ, et al. Effects of resistance training on neuromuscular characteristics and pacing during 10-km running time trial. *European journal of applied physiology.* 2015; 115(7):1513-1522.
293. Christou M, Smilios I, Sotiropoulos K, Volaklis K, Pilianidis T, Tokmakidis SP. Effects of resistance training on the physical capacities of adolescent soccer players. *Journal of Strength and Conditioning Research.* 2006; 20(4):783-791.
294. Glowacki SP, Martin SE, Maurer A, Baek W, Green JS, Crouse SF. Effects of resistance, endurance, and concurrent exercise on training outcomes in men. *Medicine and science in sports and exercise.* 2004; 36(12):2119-2127.
295. Kramer JB, Stone MH, O'Bryant HS, Conley MS, Johnson RL, Nieman DC, Honeycutt DR, Hoke TP. Effects of single vs. multiple sets of weight training: impact of volume, intensity, and variation. *Journal of Strength and Conditioning Research*. 1997; 11:143-7.
